# PrkA controls peptidoglycan biosynthesis through the essential phosphorylation of ReoM

**DOI:** 10.7554/eLife.56048

**Published:** 2020-05-29

**Authors:** Sabrina Wamp, Zoe J Rutter, Jeanine Rismondo, Claire E Jennings, Lars Möller, Richard J Lewis, Sven Halbedel

**Affiliations:** 1 FG11 - Division of Enteropathogenic bacteria and Legionella, Robert Koch InstituteWernigerodeGermany; 2 Institute for Cell and Molecular Biosciences, Medical School, University of NewcastleNewcastle upon TyneUnited Kingdom; 3 Department of General Microbiology, GZMB, Georg-August-Universität GöttingenGöttingenGermany; 4 Newcastle Drug Discovery, Northern Institute for Cancer ResearchNewcastle upon TyneUnited Kingdom; 5 ZBS 4 - Advanced Light and Electron Microscopy, Robert Koch InstituteBerlinGermany; University of the WitwatersrandSouth Africa; University of California, BerkeleyUnited States

**Keywords:** pasta-domain, serine/threonine protein kinase, peptidoglycan, protein degradation, ClpCP protease, *B. subtilis*, Other

## Abstract

Peptidoglycan (PG) is the main component of bacterial cell walls and the target for many antibiotics. PG biosynthesis is tightly coordinated with cell wall growth and turnover, and many of these control activities depend upon PASTA-domain containing eukaryotic-like serine/threonine protein kinases (PASTA-eSTK) that sense PG fragments. However, only a few PG biosynthetic enzymes are direct kinase substrates. Here, we identify the conserved ReoM protein as a novel PASTA-eSTK substrate in the Gram-positive pathogen *Listeria monocytogenes*. Our data show that the phosphorylation of ReoM is essential as it controls ClpCP-dependent proteolytic degradation of the essential enzyme MurA, which catalyses the first committed step in PG biosynthesis. We also identify ReoY as a second novel factor required for degradation of ClpCP substrates. Collectively, our data imply that the first committed step of PG biosynthesis is activated through control of ClpCP protease activity in response to signals of PG homeostasis imbalance.

## Introduction

The cell wall of Gram-positive bacteria is a complicated three-dimensional structure that engulfs the cell as a closed sacculus. The main component of bacterial cell walls is peptidoglycan (PG), a network of glycan strands crosslinked together by short peptides (Vollmer et al., 2008a). PG biosynthesis starts with the conversion of UDP-Glc*N*Ac into lipid II, a disaccharide pentapeptide that is ligated to a membrane-embedded bactoprenol carrier lipid (Typas et al., 2012). This monomeric PG precursor is then flipped from the inner to the outer leaflet of the cytoplasmic membrane by MurJ- and Amj-like enzymes called flippases (Ruiz, 2008; Sham et al., 2014; Meeske et al., 2015). Glycosyltransferases belonging either to the bifunctional penicillin binding proteins (PBPs) or the SEDS (shape, elongation, division and sporulation) family then transfer the disaccharide pentapeptides to growing PG strands, which are finally crosslinked by a transpeptidation reaction catalysed by bifunctional (class A) or monofunctional (class B) PBPs (Sauvage et al., 2008; Meeske et al., 2016; Emami et al., 2017; Taguchi et al., 2019). Numerous hydrolytic or PG-modifying enzymes are also required to adapt the sacculus to the morphological changes that occur during bacterial cell growth and division (Vollmer et al., 2008b; Uehara and Bernhardt, 2011) or to alter its chemical properties for instance for immune evasion (Moynihan et al., 2014). A suite of regulators ensure that spatiotemporal control of PG synthesis is balanced against PG hydrolysis in cycles of bacterial growth and division (Booth and Lewis, 2019).

The activity of several key enzymes along the PG biosynthetic pathway is regulated by PASTA (PBP and serine/threonine kinase associated) domain-containing eukaryotic-like serine/threonine protein kinases (PASTA-eSTKs) (Dworkin, 2015; Manuse et al., 2016; Egan et al., 2017). These membrane-integral enzymes comprise a cytoplasmic kinase domain linked to several extracellular PASTA domains (Manuse et al., 2016). These proteins are stimulated by free muropeptides and lipid II (that accumulate during damage and turnover of PG) on interaction with their PASTA domains (Mir et al., 2011; Hardt et al., 2017; Kaur et al., 2019). PknB, a representative PASTA-eSTK from *Mycobacterium tuberculosis*, phosphorylates GlmU, a bifunctional uridyltransferase/acetyltransferase important for synthesis of UDP-Glc*N*Ac, and in so doing reduces GlmU activity (Parikh et al., 2009). *M. tuberculosis* MviN, a MurJ-like flippase, is also a substrate of PknB and, in its phosphorylated state, P-MviN is inhibited by its binding partner, FhaA (Gee et al., 2012). *M. tuberculosis* PknB also phosphorylates both the class A PBP PonA1 (Kieser et al., 2015) and the amidase-like protein CwlM, which is essential for growth (Deng et al., 2005; Boutte et al., 2016; Turapov et al., 2018). CwlM is membrane-associated and interacts with MurJ to control lipid II export (Turapov et al., 2018). However, when phosphorylated, P-CwlM re-locates from the membrane to the cytoplasm (Turapov et al., 2018) where it allosterically activates MurA 20–40-fold (Boutte et al., 2016). MurA catalyzes the first committed step of PG biosynthesis by transferring an enoylpyruvate moiety to UDP-Glc*N*Ac; MurA is essential in *M. tuberculosis* and in many other bacterial species tested (Brown et al., 1995; Kock et al., 2004; Griffin et al., 2011; Rismondo et al., 2017). Finally, the *Listeria monocytogenes* PASTA-eSTK, PrkA, phosphorylates YvcK, which is required for cell wall homeostasis in a so far unknown way (Pensinger et al., 2016).

Numerous additional proteins acting to coordinate cell wall biosynthesis with cell division are substrates of PASTA-eSTKs in other Gram-positive bacteria (Manuse et al., 2016), including the late cell division protein GpsB of *Bacillus subtilis* (Macek et al., 2007; Pompeo et al., 2015). We have shown previously that GpsB from *L. monocytogenes* is important for the last two steps of PG biosynthesis, *i. e.* transglycosylation and transpeptidation, by providing an assembly platform for the class A PBP, PBP A1 (Rismondo et al., 2016; Cleverley et al., 2016; Cleverley et al., 2019; Halbedel and Lewis, 2019), and this adaptor function of GpsB is maintained in at least *B. subtilis* and *Streptococcus pneumoniae* (Cleverley et al., 2019). An *L. monocytogenes* Δ*gpsB* mutant is impaired in PG biosynthesis and cannot grow at elevated temperatures (Rismondo et al., 2016), but this phenotype is readily corrected by a suppressor mutation, which mapped to *clpC* (Rismondo et al., 2017). ClpC is the ATPase subunit of the ClpCP protease that degrades substrate proteins upon heat stress (Molière and Turgay, 2009). MurA (*aka* MurAA in *B. subtilis*) is a ClpCP substrate in both *B. subtilis* and *L. monocytogenes* (Kock et al., 2004; Rismondo et al., 2017) and strongly accumulates in a *L. monocytogenes* Δ*clpC* mutant (Rismondo et al., 2017). Thus, a deficiency in the final two enzymatic steps of PG biosynthesis in the absence of GpsB is corrected by mutations in *clpC* that increase the amount of the first enzyme of the same PG biosynthetic pathway.

We here have isolated further *gpsB* suppressor mutations affecting previously unstudied *Listeria* genes. We demonstrate that these proteins control the ClpCP-dependent degradation of MurA in a PrkA-dependent and hitherto unprecedented manner. One of them is phosphorylated by PrkA and this phosphorylation is essential. Our results represent the first molecular link between PrkA-dependent protein phosphorylation and control of PG production in low G/C Gram-positive bacteria and explain how PG biosynthesis is adjusted in these bacteria to meet PG production and repair needs.

## Results

### *gpsB* suppressor mutations in the *lmo1503* (*reoM*) and *lmo1921* (*reoY*) genes

**Figure 1.**
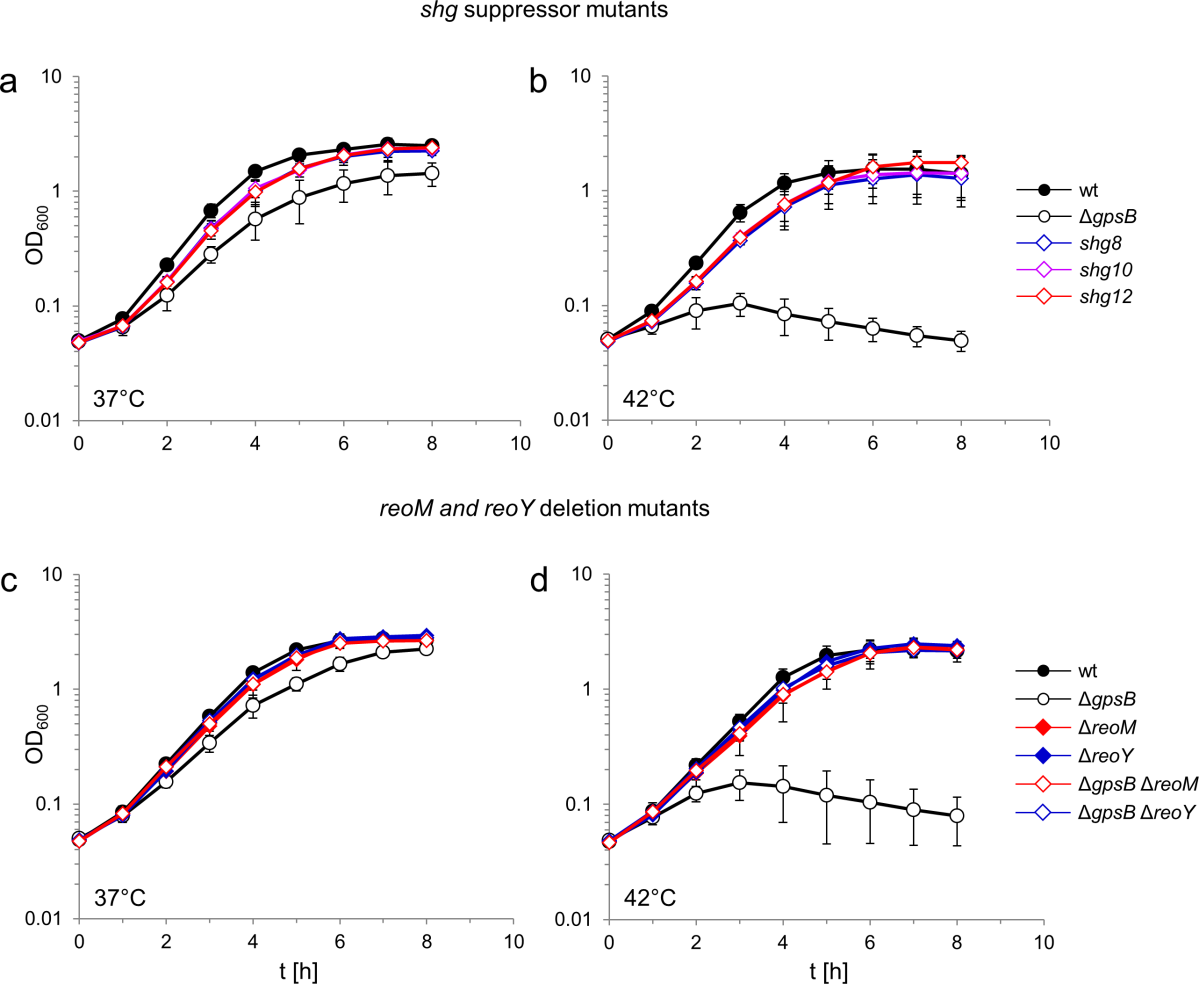
Suppression of the growth defects of a *L. monocytogenes* Δ*gpsB* mutant by *reoM* and *reoY* mutations. (**A–B**) Effect of suppressor mutations on growth of the Δ*gpsB* mutant. Growth of *L. monocytogenes* strains EGD-e (wt), LMJR19 (Δ*gpsB*), *shg8* (Δ*gpsB reoY* H87Y), *shg10* (Δ*gpsB reoY* TAA74) and *shg12* (Δ*gpsB reoM* RBS mutation) in BHI broth at 37°C (**A**) and 42°C (**B**). (**C–D**) Effect of Δ*reoM* and Δ*reoY* deletions on growth of *L. monocytogenes.* Growth of *L. monocytogenes* strains EGD-e (wt), LMJR19 (Δ*gpsB*), LMSW30 (Δ*reoM*), LMSW32 (Δ*reoY*), LMJR137 (Δ*gpsB* Δ*reoM*) and LMJR120 (Δ*gpsB* Δ*reoY*) in BHI broth was recorded at 37°C (**C**) and 42°C (**D**). All growth experiments were performed three times and average values and standard deviations are shown.

A *L. monocytogenes* Δ*gpsB* mutant is unable to replicate at 42°C, but readily forms suppressors correcting this defect (Rismondo et al., 2017). Previously isolated *gpsB* suppressors carried a mutation in the *clpC* gene, important for the stability of the UDP-*N*-acetylglucosamine 1-carboxyvinyltransferase MurA (Rismondo et al., 2017). We have characterised three more *shg* (suppression of heat sensitive growth) suppressor mutants (*shg8*, *shg10* and *shg12*) isolated from a Δ*gpsB* mutant incubated on a BHI agar plate at 42°C. These three *shg* strains grew as fast as the wild type when cultivated at 37°C or 42°C, whereas the parental Δ*gpsB* mutant grew at a reduced rate at 37°C and did not grow at 42°C (Figure 1A–B), as shown previously (Rismondo et al., 2016).

**Figure 1—figure supplement 1.**
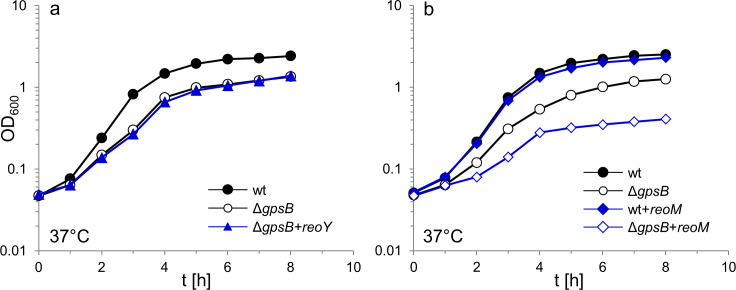
Overexpression of *reoM* but not *reoY* affects growth of the *L. monocytogenes* Δ*gpsB* mutant. (**A**) Growth of *L. monocytogenes* strains EGD-e (wt), LMJR19 (Δ*gpsB*) and LMJR106 (Δ*gpsB+reoY*) in BHI broth containing 1 mM IPTG at 37°C. (**B**) Growth of strains EGD-e (wt), LMJR19 (Δ*gpsB*), LMJR102 (wt+*reoM*) and LMJR96 (Δ*gpsB+reoM*) in BHI broth containing 1 mM IPTG at 37°C. The experiments were performed twice and one representative result is shown.

**Figure 1—figure supplement 2.**
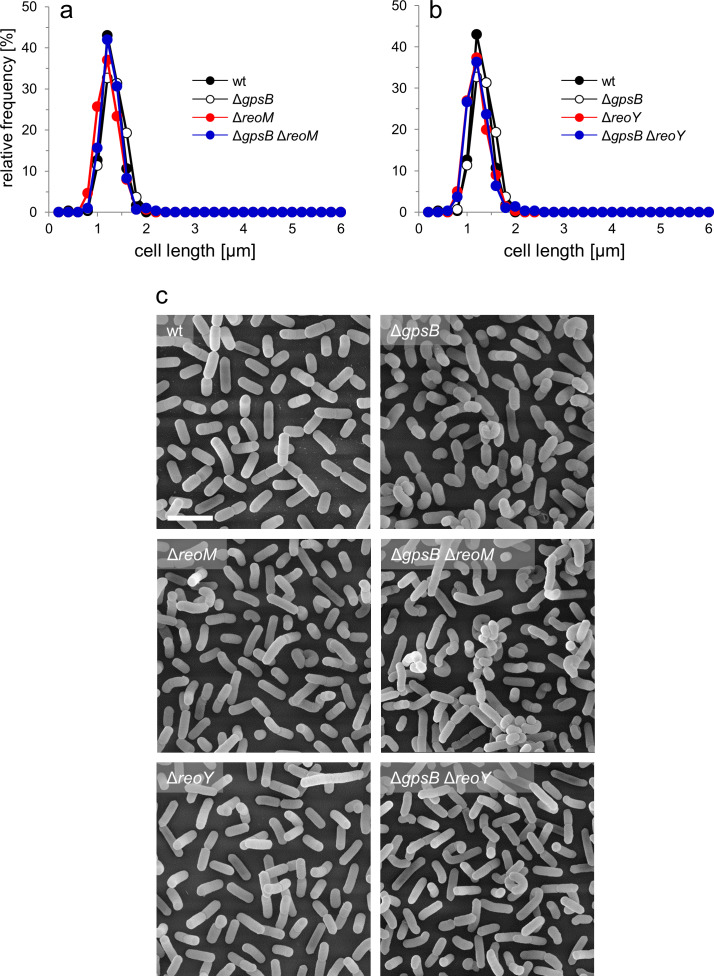
Effect of *reoM* and *reoY* deletions on cell morphology. Strains EGD-e (wt), LMJR19 (Δ*gpsB*), LMSW30 (Δ*reoM*), LMSW32 (Δ*reoY*), LMJR137 (Δ*gpsB* Δ*reoM*), and LMJR120 (Δ*gpsB* Δ*reoY*) were grown in BHI broth at 37°C to mid-logarithmic growth phase and cell lengths were measured after nile red staining and fluorescence microscopy. Diagrams show the effect of *reoM* (**A**) and *reoY* (**B**) deletions on cell lengths of wild type and Δ*gpsB* mutant cells as cell length frequency distributions determined on 300 cells per strain from a single culture. Results of one out of three experiments are shown. (**C**) Scanning electron microscopy of the same strains as in panel A and B. Strains were grown to mid-logarithmic growth phase in BHI at 37°C and subjected to chemical fixation and subsequent electron microscopy as described in the experimental procedures section. Scale bar: 2 µm.

Sequencing of the *shg8*, *shg10* and *shg12* genomes identified one SNP in each strain that was absent from the parental Δ*gpsB* mutant. Strain *shg8* carried a mutation in the uncharacterized *lmo1921* gene (herein named *reoY*, see below) that exchanged H87 into tyrosine; the same gene was affected by the introduction of a premature stop codon after the 73^rd^ *reoY* codon in strain *shg10*. Strain *shg12* carried a mutation in the ribosomal binding site (RBS) of the *lmo1503* gene (renamed *reoM*), encoding an IreB-like protein, the function of which is not understood (Hall et al., 2013).

Whether the mutation in the RBS of *reoM* in strain *shg12* affected *reoM* expression was not clear. Therefore, the *reoM* gene was deleted from the genome of the wild type and the Δ*gpsB* mutant. While deletion of *reoM* had no effect on growth of wild type bacteria, it completely suppressed the growth defects of the Δ*gpsB* mutant at both 37°C and 42°C (Figure 1C–D). It is thus likely that the mutation in the *reoM* RBS impairs its expression. Likewise, deletion of *reoY* completely restored growth of the Δ*gpsB* mutant at both temperatures (Figure 1C–D).

Expression of an additional copy of *reoM* impaired growth of the Δ*gpsB* mutant without affecting the growth of wild type bacteria, whilst expression of a second copy of *reoY* had no effect (Figure 1—figure supplement 1A,B). The expression of *reoM* is thus inversely correlated with the growth of the Δ*gpsB* mutant. Finally, the physiology of the Δ*reoM* and Δ*reoY* mutants was examined; their cell lengths were wild type-like and unaffected by the presence or absence of *gpsB*, suggesting the absence of cell division defects in the Δ*reoM* or Δ*reoY* mutants (Figure 1—figure supplement 2A,B). Scanning electron micrographs of Δ*reoM* and Δ*reoY* single mutants revealed that these bacteria had a normal rod-shape, but that the Δ*gpsB* Δ*reoM* and Δ*gpsB* Δ*reoY* double mutants were partially bent (Figure 1—figure supplement 2C), implying the presence of some shape maintenance defects along the lateral cell cylinders.

### ReoM and ReoY affect the stability of MurA

**Figure 2.**
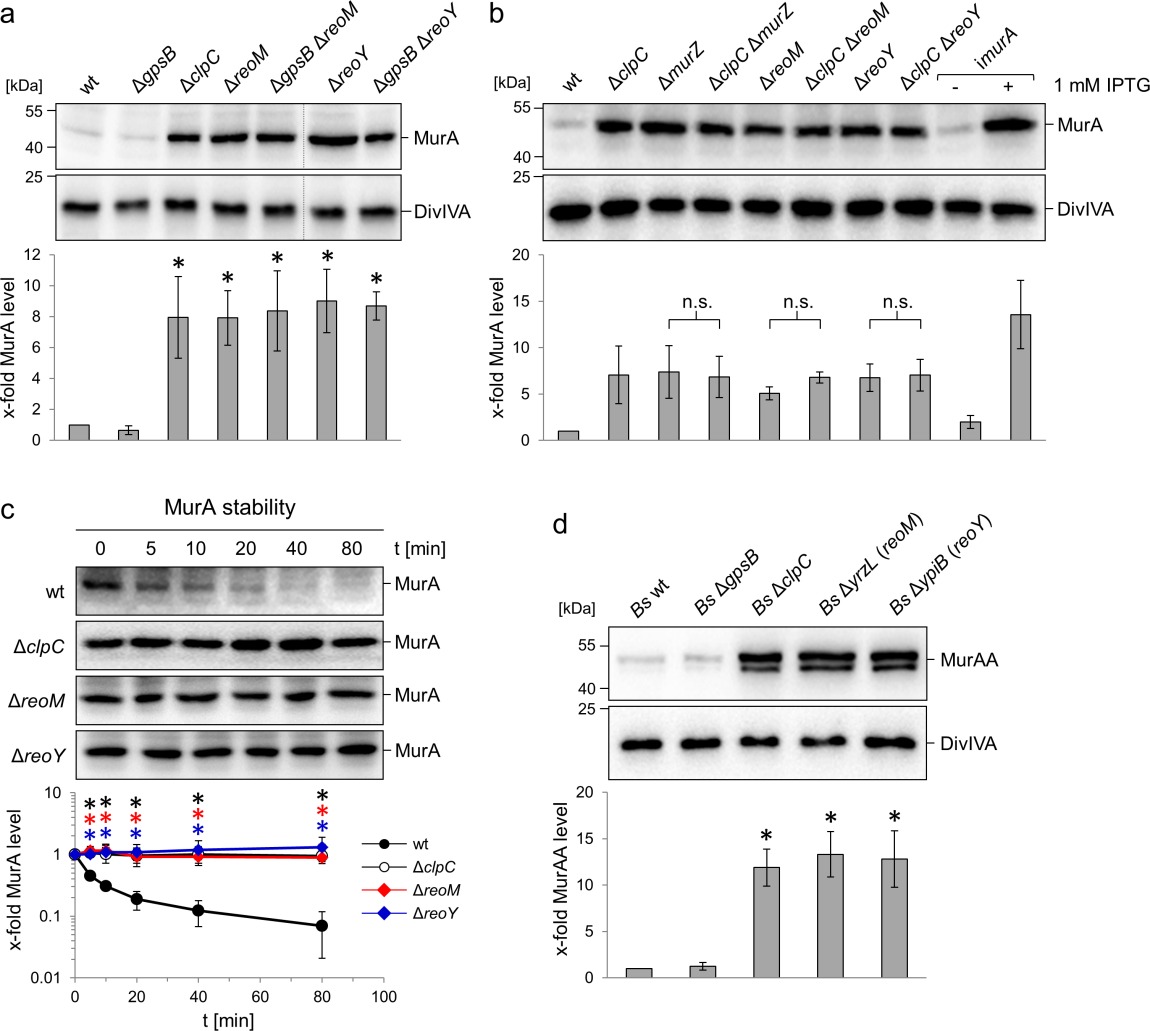
Effect of the *reoM, reoY* and *clpC* genes on levels of MurA in *L. monocytogenes* and MurAA in *B. subtilis.* (**A**) Effect of *reoM* and *reoY* deletions (single or when combined with *gpsB* deletion) on MurA (above) and DivIVA levels (middle) in *L. monocytogenes* strains EGD-e (wt), LMJR19 (Δ*gpsB*), LMSW30 (Δ*reoM*), LMSW32 (Δ*reoY*), LMJR137 (Δ*gpsB* Δ*reoM*) and LMJR120 (Δ*gpsB* Δ*reoY*) and quantification of MurA levels (below). Strain LMJR138 (Δ*clpC*) was included for comparison. Non-relevant lanes were excised from the blots (dotted lines). Average values ± standard deviations were shown (n = 3). Statistically significant differences compared to wild type are marked by asterisks (p<0.05, *t*-test). (**B**) Effect of *reoM*, *reoY* and *murZ* deletions when combined with *clpC* deletion on MurA (above) and DivIVA levels (middle) in *L. monocytogenes* strains EGD-e (wt), LMJR138 (Δ*clpC*), LMJR104 (∆*murZ*), LMJR171 (Δ*clpC* Δ*murZ*), LMSW30 (Δ*reoM*), LMSW50 (Δ*clpC* Δ*reoM*), LMSW32 (Δ*reoY*) and LMSW51 (Δ*clpC* Δ*reoY*) and quantification of MurA levels (below). Strain LMJR123 (i*murA, *i - is used to denote IPTG-dependent alleles) grown in the presence or absence of IPTG was included for comparison. Average values and standard deviations were shown (n = 3) and n. s. means not significant (p<0.05, *t*-test). (**C**) Western blots following MurA degradation in vivo. *L. monocytogenes* strains EGD-e (wt), LMJR138 (Δ*clpC*), LMSW30 (Δ*reoM*) and LMSW32 (Δ*reoY*) were grown to an OD_600_ of 1.0 and 100 µg/ml chloramphenicol was added. Samples were taken before chloramphenicol addition and after several time intervals to analyse MurA levels. MurA signals were quantified by densitometry and average values and standard deviations are shown (n = 3). Statistically significant differences are marked with asterisks (p<0.05, *t*-test). (**D**) Effect of the *reoM* and *reoY* homologues *yrzL* and *ypiB*, respectively, on MurAA (above) and DivIVA levels (middle) of *B. subtilis* and quantification of MurAA levels (below). Strains BKE00860 (Δ*clpC*), BKE22180 (Δ*gpsB*), BKE22580 (Δ*ypiB*/*reoY*) and BKE27400 (Δ*yrzL*/*reoM*) were grown to mid-logarithmic growth phase before total cellular proteins were isolated. *B. subtilis* 168 (wt) was included as control. That MurAA is detected in two isoforms had been observed earlier but the reasons for this are not known (Kock et al., 2004). Average values and standard deviations were shown (n = 3). Asterisks indicate statistically significant differences compared to wild type (p<0.05, *t*-test).

Suppression of the Δ*gpsB* phenotype can be achieved by the accumulation of MurA (Rismondo et al., 2017). Consequently, MurA levels were determined in Δ*reoM* and Δ*reoY* mutant strains by western blotting. MurA accumulated by at least eight-fold in comparison to the wild type in the absence of *reoM* or *reoY* (Figure 2A), and reached similar levels to a mutant lacking *clpC*, which encodes the ATPase subunit of the ClpCP protease (Figure 2A). MurAA, the *B. subtilis* MurA homologue, is degraded by the ClpCP protease in vivo (Kock et al., 2004). In order to test whether *reoM* and *reoY* exert their effect on MurA in a ClpC-dependent manner in *L. monocytogenes*, MurA levels were determined in Δ*clpC* Δ*reoM* and Δ*clpC* Δ*reoY* double mutants. The MurA levels in Δ*clpC*, Δ*reoM* and Δ*reoY* single mutants were the same as in Δ*clpC* Δ*reoM* and Δ*clpC* Δ*reoY* double mutant strains (Figure 2B). Likewise, the MurA level in a mutant lacking *murZ*, previously shown to contribute to MurA accumulation (Rismondo et al., 2017), is not additive to the MurA level in Δ*clpC* cells (Figure 2B). Reintroduction of *reoM*, *reoY* and *murZ* into their respective single mutant backgrounds complemented their phenotypes (Figure 2—figure supplement 1A and Figure 5C below). Therefore, ReoM, ReoY and MurZ likely affect the ClpCP-dependent degradation of MurA. Combinations of Δ*reoM,* Δ*reoY* and Δ*murZ* deletions did also not exert any additive effect on accumulation of MurA (Figure 2—figure supplement 1B), further validating the conclusion that these genes all belong to the same pathway.

**Figure 2—figure supplement 1.**
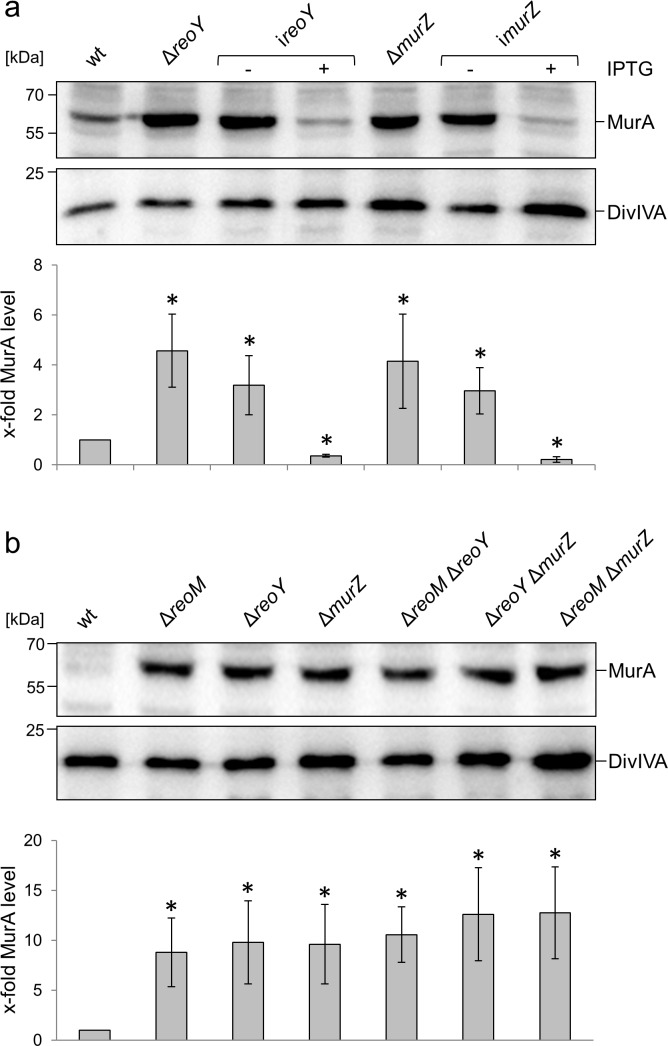
Complementation and epistasis experiments. (**A**) Complementation of Δ*reoY* and Δ*murZ* mutants. Western blot showing MurA amounts in *L. monocytogenes* strains EGD-e (wt), LMSW32 (Δ*reoY*), LMSW138 (i*reoY*), LMJR104 (Δ*murZ*) and LMSW139 (i*murZ*, top). A parallel western blot using an α-DivIVA antiserum was used as loading control (middle panel). Quantification of MurA signals by densitometry (bottom). (**B**) ReoM, ReoY and MurZ operate in the same pathway. Western blot showing MurA amounts in *L. monocytogenes* strains EGD-e (wt), LMSW30 (Δ*reoM*), LMSW32 (Δ*reoY*), LMJR104 (Δ*murZ*), LMSW117 (Δ*reoM* Δ*reoY),* LMSW118 (Δ*reoY* Δ*murZ)* and LMSW119 (Δ*reoM* Δ*murZ*, top). A parallel western blot using an α-DivIVA antiserum was used as loading control (middle panel). Quantification of MurA signals by densitometry (bottom). Strains were grown in BHI (±1 mM IPTG) at 37°C to mid-logarithmic growth phase for isolation of cellular proteins. Average values and standard deviations were calculated from three independent experiments. Asterisks indicate statistically significant differences compared to wild type (p<0.05, *t*-test).

**Figure 2—figure supplement 2.**
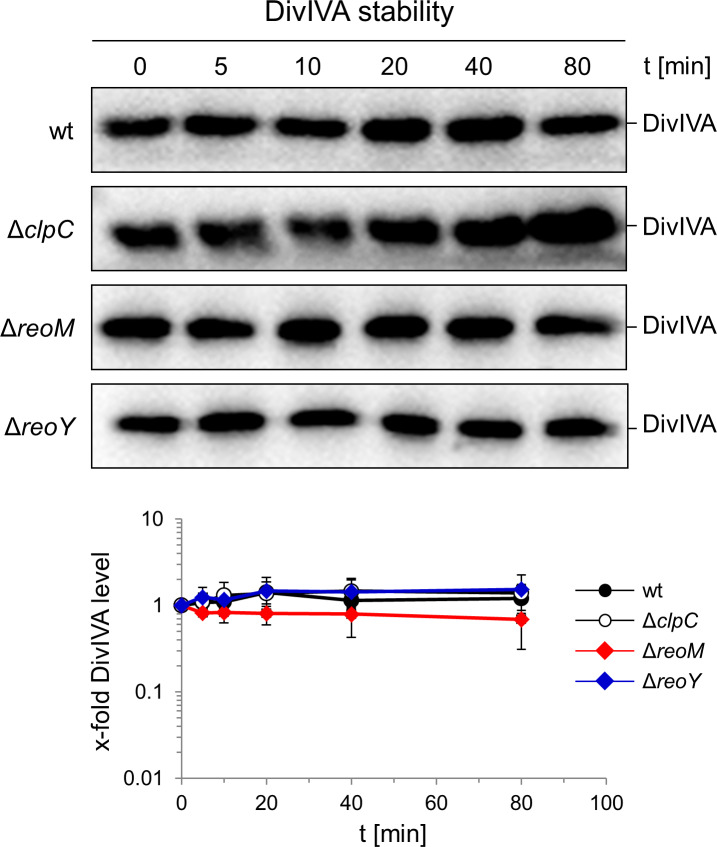
DivIVA stability in *L. monocytogenes* Δ*clpC*, Δ*reoM* and Δ*reoY* mutants. Western blots following DivIVA levels after chloramphenicol treatment. *L. monocytogenes* strains EGD-e (wt), LMJR138 (Δ*clpC*), LMSW30 (Δ*reoM*) and LMSW32 (Δ*reoY*) were grown to an OD_600_ of 1.0 and 100 µg/ml chloramphenicol was added to block protein biosynthesis. Samples were taken before chloramphenicol addition and after several time intervals to analyse DivIVA levels. DivIVA signals were quantified by densitometry and average values and standard deviations are shown (n = 3).

**Figure 2—figure supplement 3.**
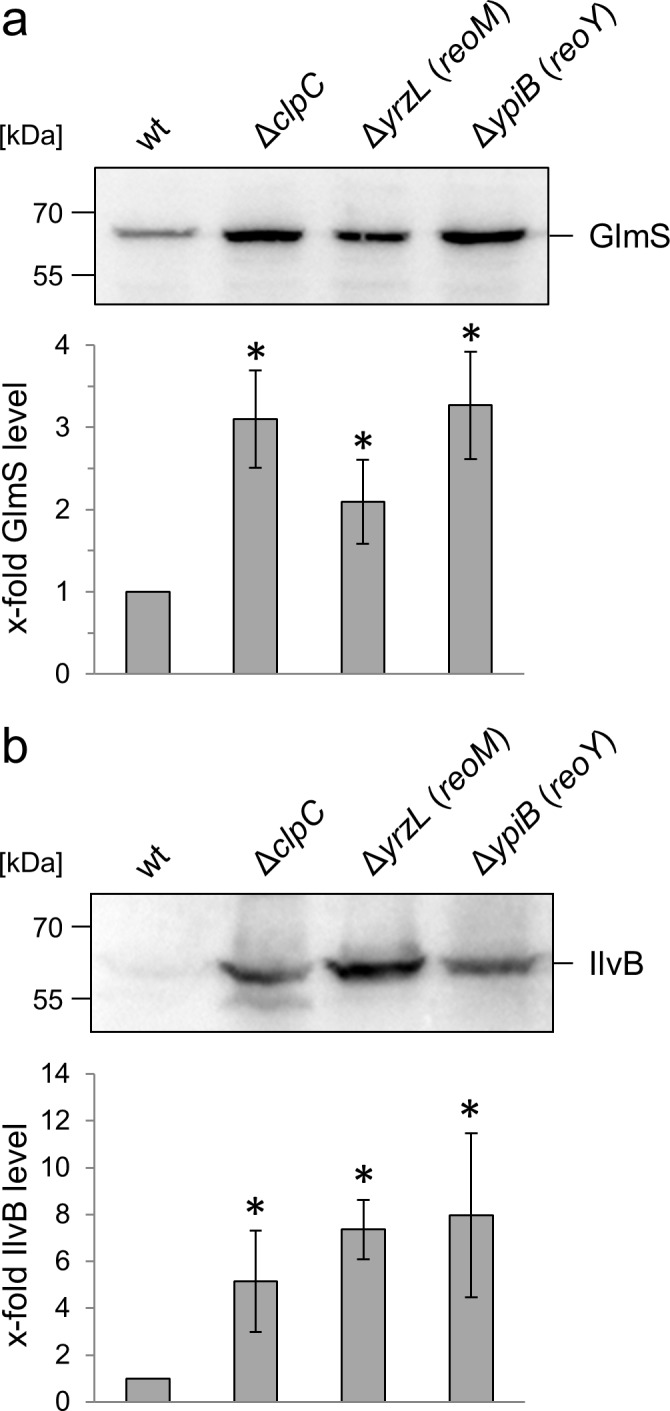
Effect of *reoM* and *reoY* deletions on accumulation of other ClpC substrates in *B. subtilis*. (**A**) Western blot showing GlmS amounts in *B. subtilis* strains 168 (wt), BKE00860 (Δ*clpC*), BKE27400 (Δ*yrzL/reoM*) and BKE22580 (Δ*ypiB/reoY*, top). Strains were grown to mid-logarithmic growth phase for isolation of cellular proteins. Quantification of GlmS signals by densitometry (bottom). (**B**) Western blot showing IlvB amounts in the same set of strains as in panel A (top). Quantification of IlvB signals by densitometry (bottom). Average values and standard deviations calculated from three independent experiments are shown. Asterisks indicate statistically significant differences (p<0.05, *t*-test). A loading control is shown in Figure 2C.

We then tested the hypothesis that ReoM and ReoY control proteolytic stability of MurA and followed MurA and DivIVA degradation over time in cells that had been treated with chloramphenicol to block protein biosynthesis. MurA was almost completely degraded in wild type cells 80 min after chloramphenicol treatment (Figure 2C), whereas DivIVA was stable (Figure 2—figure supplement 2B). By contrast, no MurA degradation was observed in mutants lacking *clpC*, *reoM* or *reoY* (Figure 2C), which together demonstrates that ReoM and ReoY are as important for MurA degradation as is ClpC.

### The effect of ReoM and ReoY on MurA levels is conserved

Homologues of the 90-residue ReoM protein are found across the entire Firmicute phylum, and include IreB, a substrate of the protein serine/threonine kinase IreK and its cognate phosphatase IreP from *Enterococcus faecalis* (Hall et al., 2013), whereas ReoY homologues are present only in the *Bacilli*. A *reoY* homologue has been identified as a Δ*ireK* suppressor in *E. faecalis* (Banla et al., 2018), but the function of the *E. faecalis reoY* and *reoM* homologues remains unknown. In *B. subtilis*, ReoM corresponds to YrzL (e-value 3e^−29^) and ReoY to YpiB (4e^−61^), but neither protein has been studied thus far. To assess whether YrzL and YpiB were also crucial for control of MurAA levels in *B. subtilis*, cellular protein extracts from *B. subtilis* Δ*yrzL* and Δ*ypiB* mutants were probed by western blot (Figure 2D). MurAA accumulated by at least 12-fold in these strains in comparison to the wild type. Furthermore, the amount of MurAA was also increased by 12-fold in the Δ*clpC* mutant. Taken together, these data indicate that ReoM and ReoY functions are conserved in both species. We thus propose to rename *lmo1503* (*yrzL*) as *reoM* (regulator of MurA(A) degradation) and analogously *lmo1921* (*ypiB*) as *reoY*.

Several other ClpC substrates are known in *B. subtilis*, including the glutamine fructose-6-phosphate transaminase GlmS and the acetolactate synthase subunit IlvB (Gerth et al., 2008). The levels of both proteins were also significantly increased in *B. subtilis* Δ*reoM* and Δ*reoY* mutants (Figure 2—figure supplement 3), indicating that ReoM and ReoY are required for degradation of ClpC substrates in general.

### ReoM and ReoY contribute to PG biosynthesis

**Figure 3.**
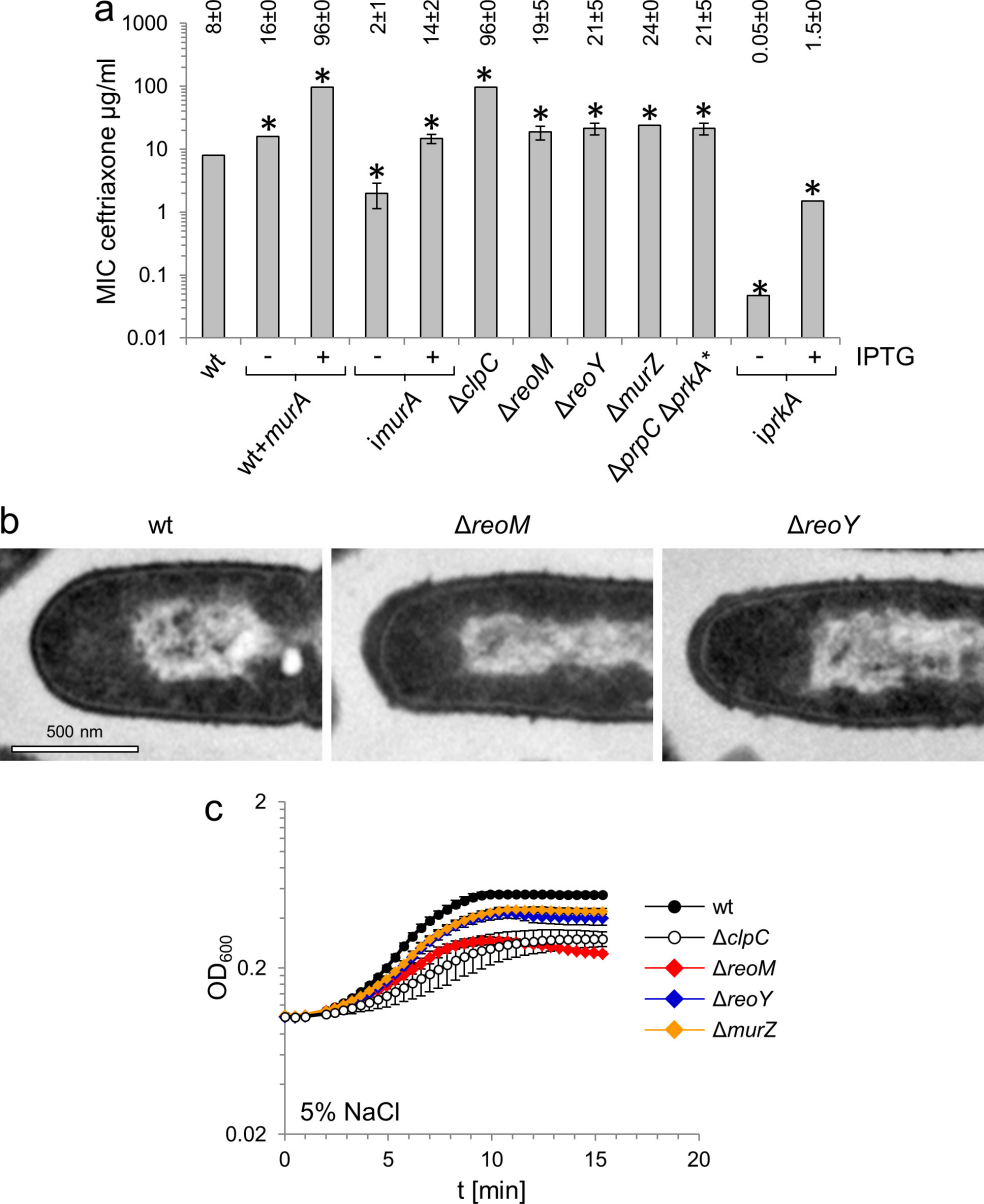
MurA accumulation affects peptidoglycan biosynthesis and salt sensitivity. (**A**) Minimal inhibitory concentrations (MIC) for ceftriaxone of mutants with altered MurA accumulation. Average values and standard deviations are calculated from three independent experiments and given above the panel. Asterisks indicate statistically significant differences compared to wild type (p<0.05, *t*-test). Please note that the i*prkA* strain showed residual growth on BHI agar plates not containing IPTG, even though it required IPTG for growth in BHI broth. (**B**) Transmission electron microscopy of ultrathin sections of fixed whole cells of *L. monocytogenes* wildtype, Δ*reoM* and Δ*reoY* mutants. *L. monocytogenes* strains EGD-e (wt), LMSW30 (Δ*reoM*) and LMSW32 (Δ*reoY*) were grown to mid-logarithmic growth phase in BHI broth at 37°C and subjected to chemical fixation and subsequent electron microscopy as described in the experimental procedures section. (**C**) Salt sensitive growth of mutants with altered MurA accumulation. *L. monocytogenes* strains EGD-e (wt), LMJR138 (Δ*clpC*), LMSW30 (Δ*reoM*), LMSW32 (Δ*reoY*) and LMJR104 (∆*murZ*) were grown in BHI broth containing 5% NaCl at 37°C. Average values and standard deviations are calculated from three independent experiments.

In order to test whether MurA accumulation affected PG production, we tested the effect of enhanced MurA levels on resistance of *L. monocytogenes* against the cephalosporin antibiotic ceftriaxone. Artificial overproduction of MurA in strain LMJR116, which carries an IPTG-inducible *murA* gene in addition to the native copy on the chromosome, lead to a 12-fold increase of ceftriaxone resistance, while MurA depletion lowered ceftriaxone resistance (Figure 3A). MurA levels are thus directly correlated with PG production, presumably leading to stimulation or impairment of PG biosynthesis during overproduction and depletion, respectively. In good agreement with the overproduction of MurA, ceftriaxone resistance of the Δ*clpC* mutant increased to the same degree as when MurA was overproduced (Figure 3A). Ceftriaxone resistance of Δ*reoM*, Δ*reoY* and Δ*murZ* mutants increased two- to three-fold (Figure 3A); this intermediate resistance level is probably explained by the presence of functional ClpCP in these strains. Nevertheless, these observations are consistent with a function of ReoM, ReoY and MurZ as regulators of ClpCP-dependent MurA degradation. In good agreement with this concept of stimulated PG biosynthesis during MurA accumulation, we observed thicker PG layers at the cell poles of Δ*reoM* and Δ*reoY* mutants, which also have more uneven PG layers along their lateral wall, whereas both phenomena were not observed in wild type cells (Figure 3B; Figure 3—figure supplement 1). Moreover, Δ*reoM*, Δ*reoY* and Δ*murZ* mutants showed salt-sensitive growth (Figure 3C), which is a known phenotype of the *L. monocytogenes* Δ*clpC* mutant (Rouquette et al., 1996). Salt sensitivity of the Δ*reoM* mutant was as severe as for the Δ*clpC* mutant, whereas the Δ*reoY* and Δ*murZ* mutants showed milder phenotypes (Figure 3C). Taken together, these results indicate that modulation of MurA levels effectively controls PG biosynthesis and also demonstrate that ReoM, ReoY and MurZ play an important role in its regulation.

**Figure 3—figure supplement 1.**
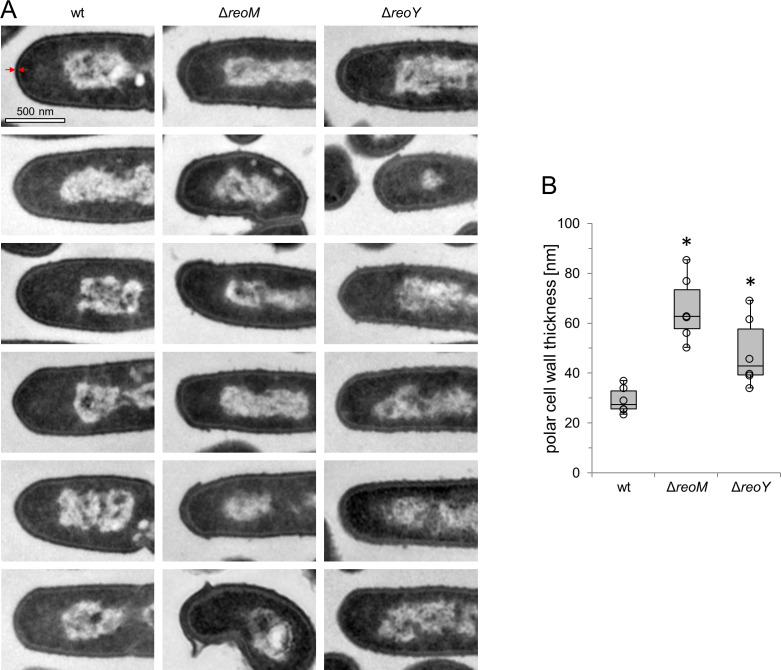
ReoM and ReoY affect thickness of polar peptidoglycan. (**A**) Representative micrographs from transmission electron microscopy images of ultrathin sections of fixed whole cells of L. monocytogenes wildtype, ΔreoM and ΔreoY mutants. (**B**) Quantification of peptidoglycan (PG) thickness at cell poles. Thickness of the PG layer at the longitudinal axis of the cell was measured as indicated by the red arrows in panel A. The boxplot shows results from 6 cells per strain. Asterisks mark statistically significant differences (P<0.05, t-test).

### Phosphorylation and dephosphorylation of ReoM by PrkA and PrpC in vitro

**Figure 4.**
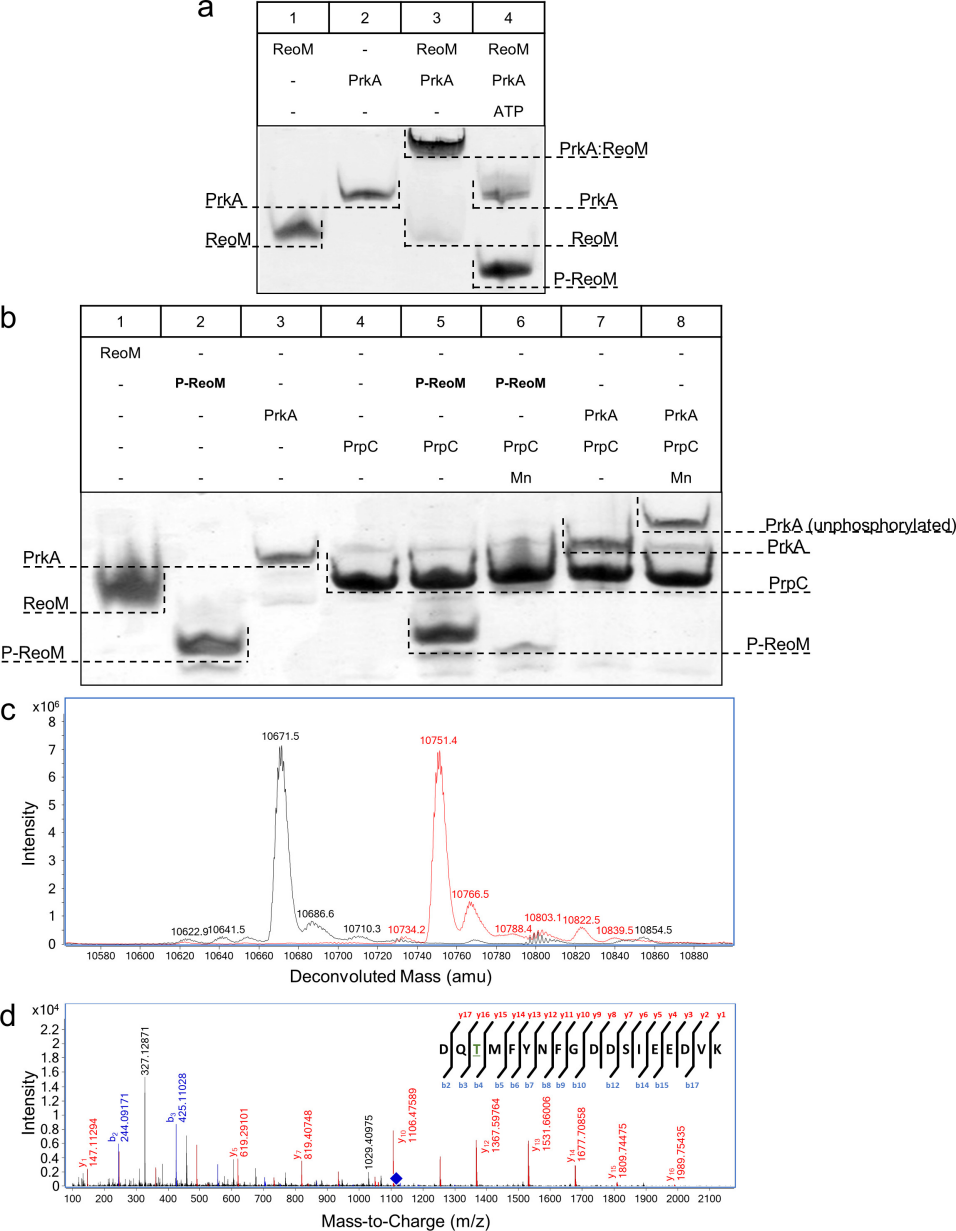
The PrkA/PrpC pair controls the phosphorylation status of ReoM. (**A–B**) Non-denaturing, native PAGE analysis of the phosphorylation (**A**) and dephosphorylation (**B**) of ReoM in vitro. The components of each lane in the Coomassie-stained gel are annotated above the image and the position and identity of relevant bands is marked to the side. (**C**) LC-MS analysis of intact ReoM. The deconvoluted mass spectrum for non-phosphorylated ReoM (black) is overlaid over the equivalent spectrum for mono-phosphorylated ReoM, P-ReoM (red). (**D**) LC-MS/MS was used to perform peptide mapping analysis that revealed that Thr7 is the sole phosphosite of ReoM. The MS/MS fragmentation spectra of the phosphorylated peptide encompassing Asp5-Lys22 is presented with *b*-ion fragmentation in blue and *y*-ion fragmentation shown in red, whilst the precursor ion (m/z 1116.86, z = 2+) is represented by a blue diamond.

PrkA (encoded by *lmo1820*) and PrpC (*lmo1821*) are the *L. monocytogenes* homologues of *E. faecalis* IreK and IreP, respectively. Consequently, the pairwise interactions and biochemical properties of ReoM, the PrkA kinase domain (PrkA-KD) and the cognate phosphatase PrpC were investigated. All isolated proteins electrophoresed as single species in non-denaturing PAGE (lanes 1, 2, Figure 4A; lanes 1–4, Figure 4B). When ReoM was incubated with PrkA-KD, in the absence of ATP, a slower migrating species was observed and the individual bands corresponding to ReoM and PrkA-KD disappeared indicating that the slower migrating species was a ReoM:PrkA-KD complex (lane 3, Figure 4A). When ReoM was incubated with PrkA-KD and Mg/ATP under the same conditions, free PrkA-KD was observed but no bands equivalent to ReoM and the ReoM:PrkA-KD complex remained; instead a new species was present, migrating faster in the gel than ReoM (lane 4, Figure 4A), which is likely to be phosphorylated ReoM (P-ReoM). Intact protein liquid chromatography-mass spectrometry (LC-MS) analysis of ReoM isolated from PrkA-KD after phosphorylation revealed the addition of 79.9 Da in comparison to the mass of ReoM (10671.5 Da), which corresponds to the formation of a singly-phosphorylated ReoM product of 10751.4 Da (Figure 4C, Figure 4—figure supplement 1). MS/MS spectra obtained during peptide mass fingerprinting were also consistent with one phosphorylation event per protein chain; one ReoM peptide, spanning residues Asp5 to Lys22 with mass of 2151.89 Da, was increased by 79.96 Da after incubation with PrkA-KD and Mg/ATP. Analysis of the *y-* and *b-* ions in the MS/MS fragmentation spectrum of this peptide was consistent only with Thr7 as the sole phosphosite in ReoM (Figure 4D). Finally, mutation of Thr7 to alanine completely abrogated the phosphorylation of ReoM by PrkA-KD when analysed by LC-MS (Figure 4—figure supplement 2).

**Figure 4—figure supplement 1.**
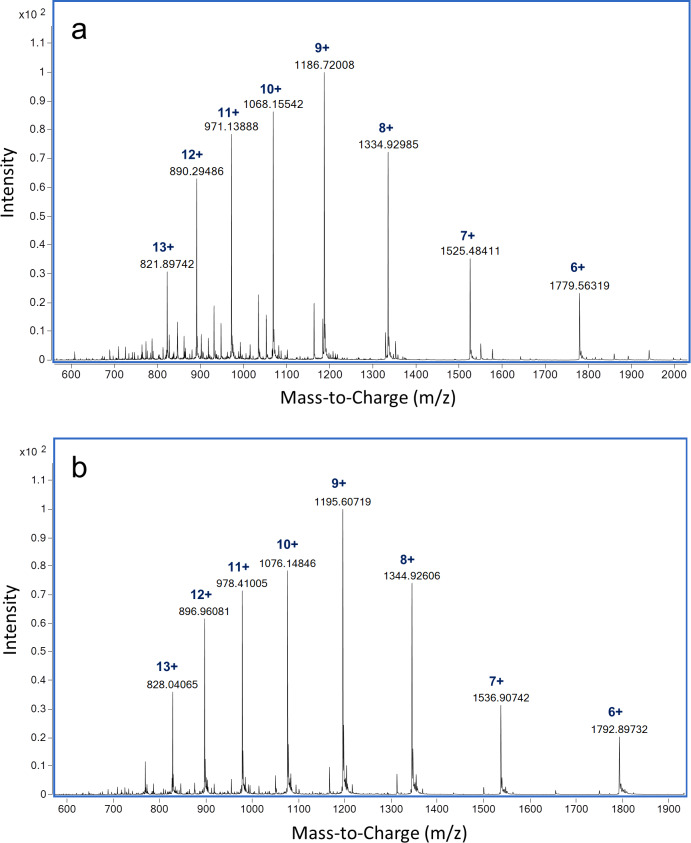
LC-MS analysis of intact ReoM. ESI-QTOF raw data for (**A**) unmodified ReoM and (**B**) mono-phosphorylated ReoM.

**Figure 4—figure supplement 2.**
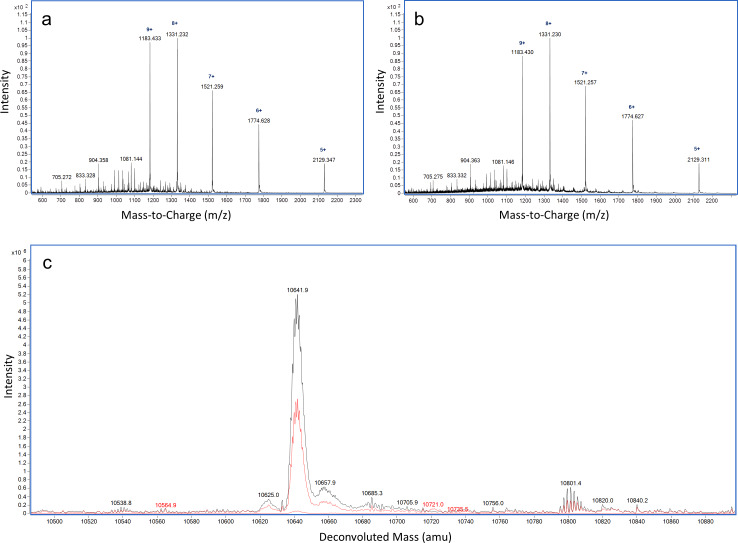
LC-MS analysis of ReoM T7A. ESI-QTOF raw data for (**A**) unmodified ReoM T7A and (**B**) ReoM T7A following incubation with PrkA-KD. (**C**) Overlaid deconvoluted mass spectrum demonstrating the lack of phosphorylation of ReoM T7A in the absence (black) and presence of PrkA-KD (red).

**Figure 4—figure supplement 3.**
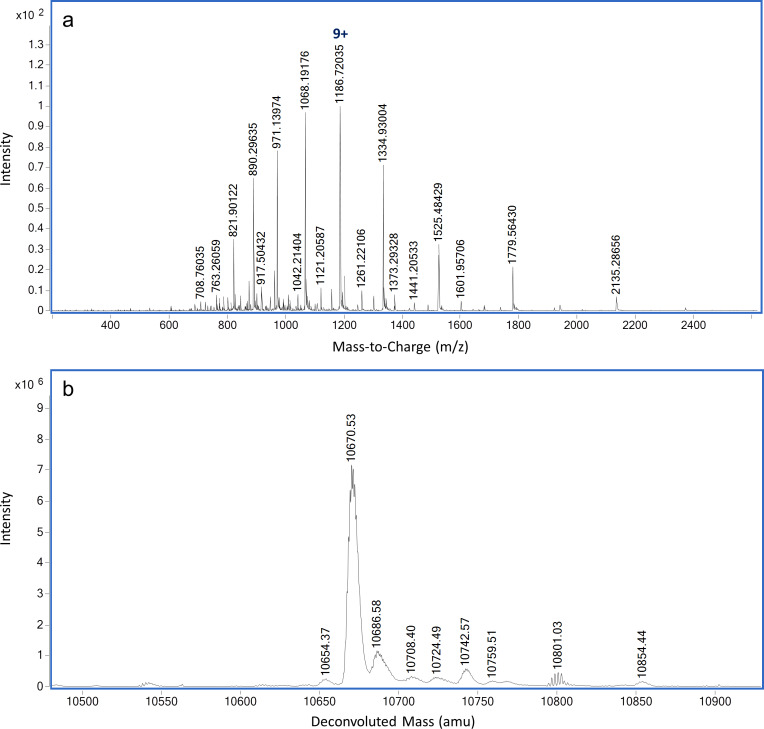
Dephosphorylation of P-ReoM by PrpC. LC-MS analysis of ReoM after PrpC incubation in the presence of manganese. (**A**) ESI-QTOF raw data and (**B**) deconvoluted mass spectrum demonstrating the presence of non-phosphorylated ReoM.

**Figure 4—figure supplement 4.**
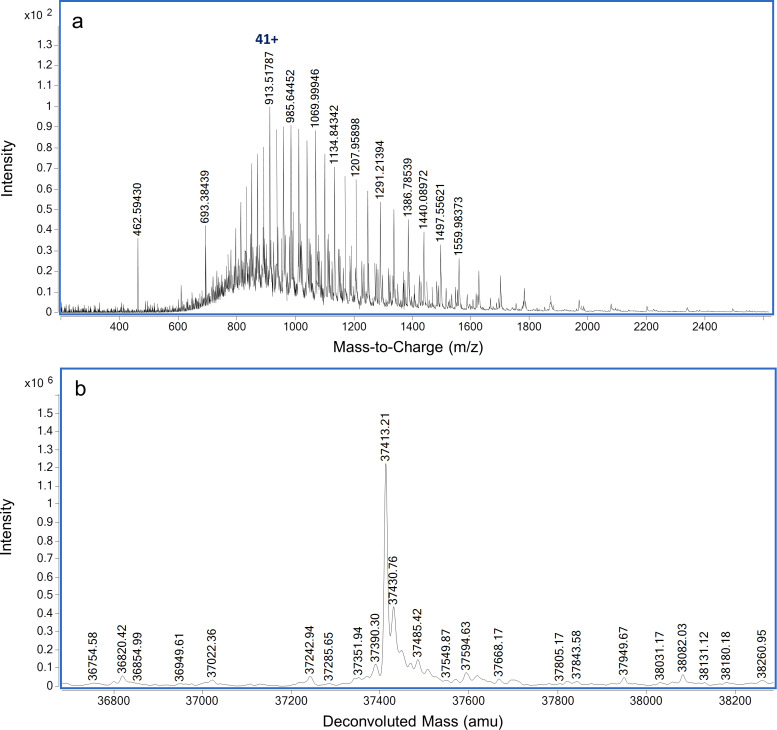
Dephosphorylation of P-PrkA-KD by PrpC. LC-MS analysis of intact PrkA-KD following incubation with PrpC (**A**) ESI-QTOF raw data and (**B**) deconvoluted mass spectrum indicating the presence of non-phosphorylated PrkA-KD.

The ability of PrpC, the partner phosphatase to PrkA in *L. monocytogenes,* to interact with and remove phosphoryl groups from PrkA-KD and P-ReoM was also tested in vitro. PrkA and purified P-ReoM were each incubated with PrpC in the absence and presence of MnCl_2_, since divalent cations are essential co-factors for the PPM phosphatase family to which PrpC belongs (Kennelly, 2001), and the products were analysed by non-denaturing PAGE. Unlike the situation with ReoM and PrkA-KD, no stable protein:protein complexes were formed either in the presence or absence of endogenous MnCl_2_ (Figure 4B). The incubation of P-ReoM with PrpC and manganese resulted in the almost complete disappearance of the band corresponding to P-ReoM (lane 6, Figure 4B) in comparison to the same reaction conducted without the addition of MnCl_2_ (lane 5, Figure 4B). The new band, corresponding to ReoM alone in lane 6, is masked by that for PrpC that migrates similarly to ReoM (lanes 1 and 4, Figure 4B) under these electrophoresis conditions. The presence of unphosphorylated ReoM and the absence of P-ReoM was confirmed by LC-MS (Figure 4—figure supplement 3). When incubated with PrpC in the presence of manganese ions, the band for PrkA-KD electrophoresed more slowly than for PrkA-KD in isolation (lanes 3 and 8, Figure 4B), indicating that PrkA-KD had been dephosphorylated by PrpC. LC-MS analysis of PrkA-KD that had been incubated with PrpC/MnCl_2_ yielded a single major species of 37,413.2 Da, consistent with the predicted mass of the expressed recombinant construct, and the absence of a peak corresponding to phosphorylated PrkA-KD, P-PrkA-KD (Figure 4—figure supplement 4). Therefore, PrkA-KD is capable of autophosphorylation even when expressed in a heterologous host, consistent with previous observations made for similar PASTA-eSTKs from other Gram-positive bacteria (Madec et al., 2003; Kristich et al., 2011). Finally, in the absence of MnCl_2_ no change in electrophoretic mobility was observed for P-PrkA-KD (lane 7, Figure 4B).

### Phosphorylation of ReoM at threonine seven is essential for viability

**Figure 5.**
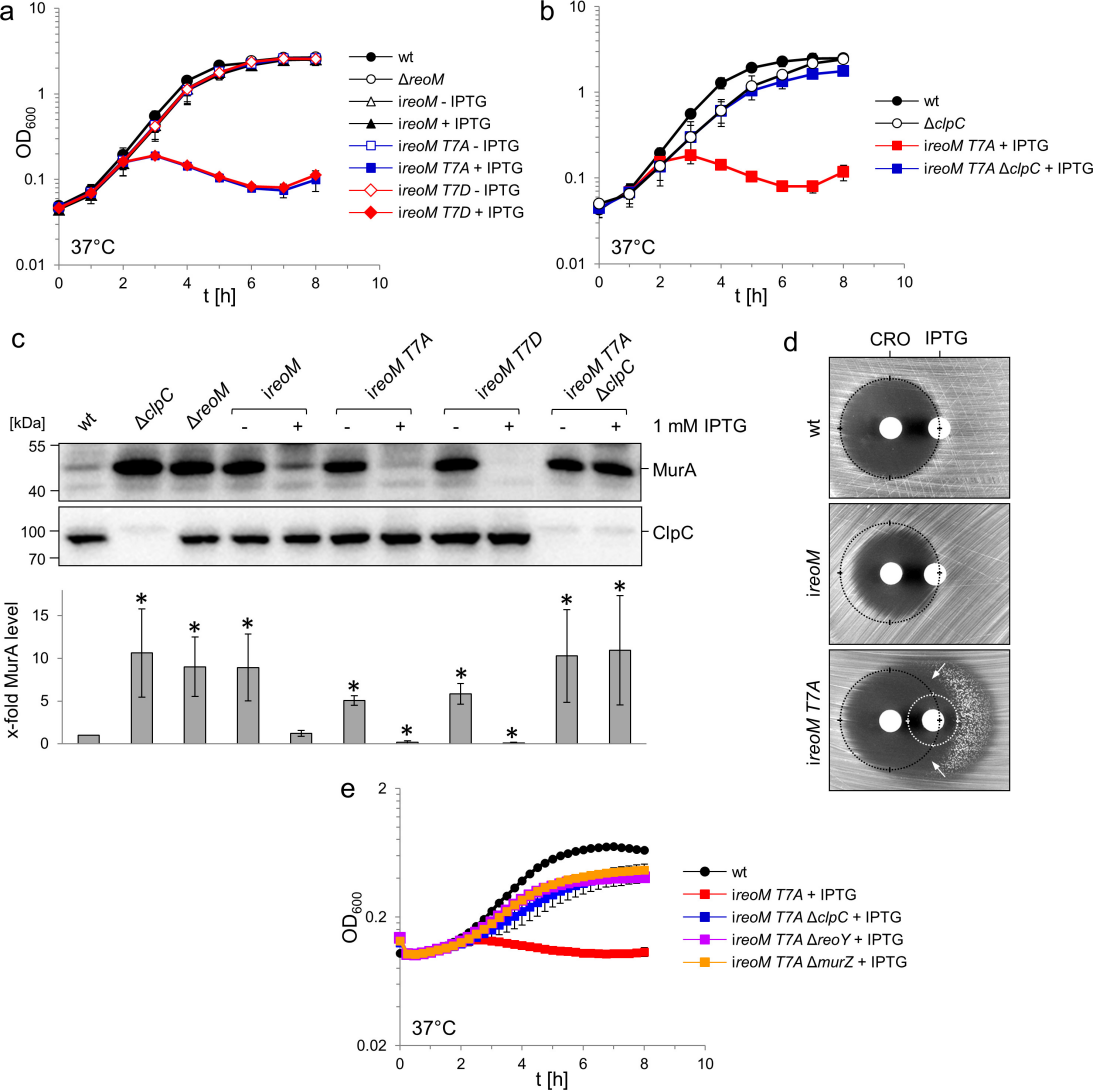
A ReoM T7A exchange affects growth and MurA levels in a ClpC-dependent manner. (**A**) Lethality of the *reoM T7A* and *reoM T7D* mutations in *L. monocytogenes. L. monocytogenes* strains EGD-e (wt), LMSW30 (Δ*reoM*), LMSW57 (i*reoM*), LMSW52 (i*reoM T7A*) and LMSW53 (i*reoM T7D*) were grown in BHI broth ±1 mM IPTG at 37°C. The experiment was repeated three times and average values and standard deviations are shown. (**B**) Suppression of *reoM T7A* lethality by deletion of *clpC. L. monocytogenes* strains EGD-e (wt), LMJR138 (Δ*clpC*), LMSW52 (i*reoM T7A*) and LMSW72 (i*reoM T7A* Δ*clpC*) were grown in BHI broth ±1 mM IPTG at 37°C. The experiment was repeated three times and average values and standard deviations are shown. (**C**) Western blot showing cellular levels of MurA (top) and ClpC (middle) in the strains included in panels A and B. For this experiment, strains were grown in BHI broth not containing IPTG at 37°C. IPTG (1 mM) was added to the cultures at an OD_600_ of 0.2 and the cells were collected 2 hr later. Quantification of MurA signals by densitometry is shown below the western blots. Average values and standard deviations calculated from three independent experiments are shown. Asterisks indicate statistically significant differences (p<0.05, *t*-test). (**D**) ReoM T7A expression sensitises *L. monocytogenes* against ceftriaxone. Synergism between ceftriaxone and IPTG in the i*reoM T7A* strain LMSW52 in a disc diffusion assay with filter discs containing 50 mg/ml ceftriaxone (CRO, left) and 1 mM IPTG (right). For comparison, wild type levels of growth inhibition by ceftriaxone are marked with black circles. Zone of growth inhibition by IPTG in the i*reoM T7A* mutant is marked with a white circle. Please note that strain LMSW52 shows hetero-resistance against IPTG (two zones of growth inhibition with different resistance levels). Arrows mark the zones of synergism between ceftriaxone and IPTG. (**E**) Contribution of ReoY and MurZ to the lethal *reoM T7A* phenotype. *L. monocytogenes* strains EGD-e (wt), LMSW52 (i*reoM T7A*), LMSW72 (i*reoM T7A* Δ*clpC*), LMSW123 (i*reoM T7A* Δ*reoY*) and LMSW124 (i*reoM T7A* Δ*murZ*) were grown in BHI broth containing 1 mM IPTG and growth at 37°C was recorded in a microplate reader. Average values and standard deviations were calculated from an experiment performed in triplicate.

PrkA phosphorylates ReoM on Thr7 and PrpC reverses this reaction in vitro; ReoM phosphorylation at Thr7 in vivo has also been observed by phosphoproteomics (Misra et al., 2011). In the absence of molecular details on the impact of Thr7 phosphorylation we determined the importance of this phosphorylation in vivo by engineering a phospho-ablative T7A exchange in an IPTG-inducible allele of *reoM* and introduced it into the Δ*reoM* mutant background. Deletion, depletion or expression of wildtype *reoM* had no effect on growth in strains LMSW30 (Δ*reoM*) and LMSW57 (i*reoM,* i - is used to denote IPTG-dependent alleles throughout the manuscript) at 37°C. Likewise, strain LMSW52 (i*reoM T7A*) grew normally in the absence of IPTG. However, the *reoM* mutant with the T7A mutation did not grow at all in the presence of IPTG, when expression of the phospho-ablative *reoM T7A* allele was induced (Figure 5A), suggesting that phosphorylation of ReoM at Thr7 is essential for the viability of *L. monocytogenes*. We next engineered a *reoM T7D* mutant to mimic the effect of Thr7 phosphorylation. However, the resulting strain was as sensitive to IPTG as the *reoM T7A* mutant (Figure 5A). Since ReoM influences the proteolytic stability of MurA, we determined the cellular amount of MurA in strains expressing the T7A/T7D variants of ReoM. For this purpose, strains LMSW57 (i*reoM*), LMSW52 (i*reoM T7A*) and LMSW53 (i*reoM T7D*) were initially cultivated in plain BHI broth. At an OD_600_ of 0.2, IPTG was added to a final concentration of 1 mM and cells were harvested 2 hr later. Strain LMSW57 (i*reoM*) showed Δ*clpC*-like MurA accumulation (around seven-fold in this experiment) when cultured in the absence of IPTG, but MurA was present at wild type levels in the presence of IPTG (Figure 5C). The strains with the T7A and T7D exchanges also accumulated MurA to a Δ*clpC*-like extent in the absence of IPTG. However, only a minor fraction of the wild type MurA levels could be detected in cells expressing the *reoM T7A* (17 ± 2%) or *reoM T7D* alleles (10 ± 2%, Figure 5C). That the *reoM T7D* mutant does not have the opposite phenotype as the *reoM T7A* mutant indicates that ReoM T7D behaves as a non-phosphorylatable protein and not as a genuine phospho-mimetic variant. The reasons for this discrepancy are currently not clear, but phospho-mimetic mutations do not work in all cases (Dephoure et al., 2013), since aspartate (and glutamate) are unfaithful chemical mimics of phosphothreonine; a similar phenomenon was also observed with phospho-mimetic replacements of Thr7 in *E. faecalis* IreB (Hall et al., 2013). Nonetheless, our data demonstrate that Thr7 in ReoM is of special importance for the proteolytic stability of MurA. In agreement with these results, IPTG was toxic for the i*reoM T7A* mutant in a disc diffusion assay and rendered this strain hypersensitive to ceftriaxone (Figure 5D).

### Lethality of the *reoM T7A* mutations depends on ClpC

That MurA is rapidly degraded in cells expressing *reoM T7A* implies that phosphorylation/dephosphorylation of ReoM at Thr7 controls ClpCP-dependent MurA degradation. MurA is an essential enzyme in *L. monocytogenes* (Rismondo et al., 2017), and stimulation of ClpCP-dependent MurA degradation in the *reoM T7A* mutant would provide an explanation for the lethality of this mutation. In order to address this possibility, we deleted *clpC* in the conditional i*reoM T7A* background. This strain grew even in the presence of IPTG, a compelling demonstration that the removal of *clpC* suppressed the lethality of the *reoM T7A* mutation (Figure 5B). MurA also accumulated to the same degree as in the Δ*clpC* mutant in this strain (Figure 5C), suggesting that inactivation of the ClpCP-dependent degradation of MurA overcame the lethal effect of the T7A mutation in *reoM* and this suggests that ClpCP acts downstream of ReoM. We next wondered whether deletion of *reoY* and *murZ* would have a similar effect and deleted these genes in the *reoM T7A* mutant. As can be seen in Figure 5E, deletion of either gene overcame the lethality of *reoM T7A*, indicating that ReoY and MurZ must also act downstream of ReoM.

### Crystal structure of ReoM, a homologue of *Enterococcus faecalis* IreB

**Figure 6.**
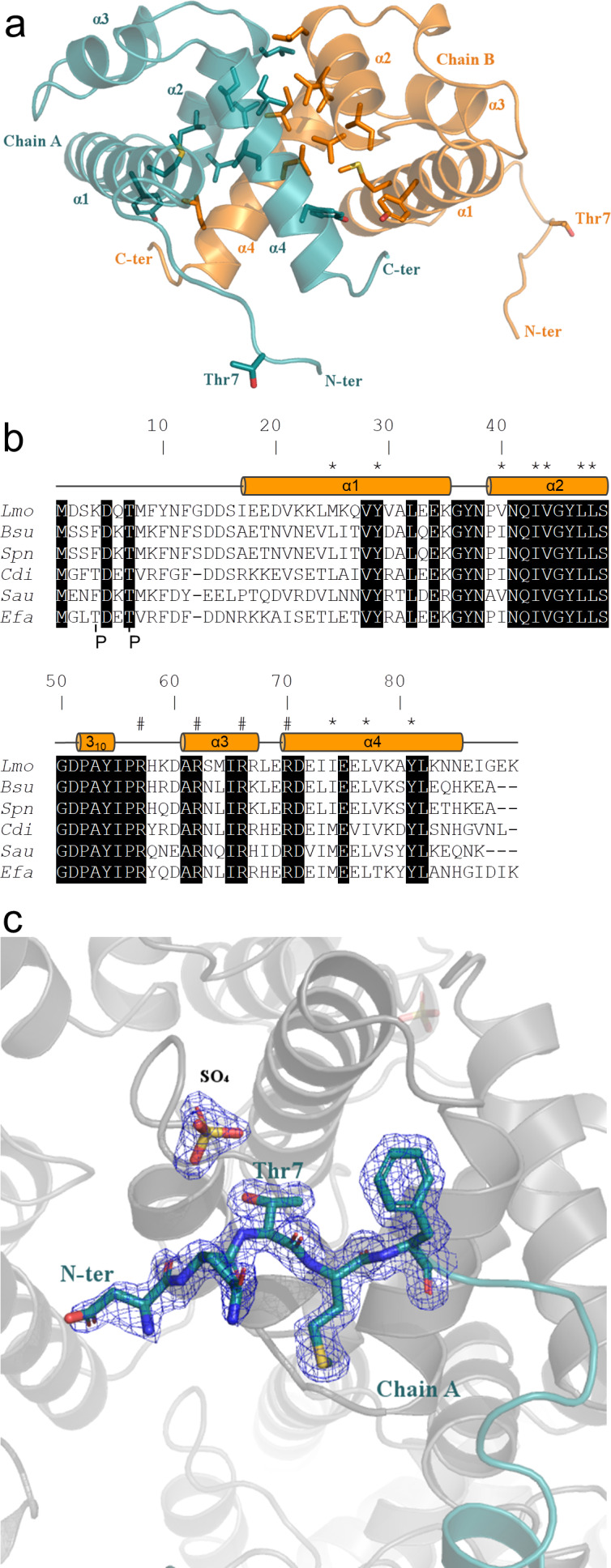
Crystal structure of ReoM. (**A**) The structure of ReoM depicted as a cartoon with each protomer in the dimer coloured separately (cyan and orange). The secondary structure elements are numbered according to their position in the amino acid sequence. Thr7 and some of the key amino acids in the dimer interface and the hydrophobic core are drawn as stick figures. (**B**) Sequence alignment of ReoM (*Lmo*) and its homologues from *Bacillus subtilis* (*Bsu*), *Streptococcus pneumoniae* (*Spn*), *Clostridium difficile* (*Cdi*) and *Staphylococcus aureus* (*Sau*) with the sequence of IreB from *Enterococcus faecalis* (*Efa*) underneath. Amino acid sequence numbers pertain to ReoM and the site of phosphorylation in ReoM (Thr7) and the twin phosphorylations in IreB (minor site: Thr4; major site: Thr7) are highlighted. Invariant amino acids are shaded black, residues in the ReoM dimer interface have an asterisk above, and the secondary structure elements are defined by cylinders above the alignment. Arginine residues mutated in this study are indicated by a hashtag above the alignment. (**C**) The final 2F_obs_-F_calc_ electron density map, contoured at a level of 0.42 e^-^/Å^3^, of the N-terminal region in the immediate vicinity of Thr7 in chain A of the ReoM dimer indicates that the protein model could be built with confidence even though this region contains no secondary structure elements.

Purified ReoM yielded crystals that diffracted to a maximum resolution of 1.6 Å. The NMR structure of IreB (PDBid 5US5) (Hall et al., 2017) was used to solve the structure of ReoM by molecular replacement (Figure 6A). The data collection and refinement statistics for the ReoM structure are summarised in Table 1. ReoM shares the same overall fold as IreB (Hall et al., 2017), each containing a compact 5-helical bundle (four standard α-helices and one single-turned 3_10_-helix between residues 52 and 54) with short loops between the secondary structure elements, which are defined above the sequence alignment in Figure 6B. Other than IreB (Hall et al., 2017), there are no structural homologues of ReoM with functional significance in the PDB. The helical bundles in both ReoM and IreB associate into homodimers with α-helices two and four from each protomer forming the majority of the homodimer interface (Figure 6A), and these residues are highlighted in Figure 6B. In agreement with the IreB structural analysis, 1200 Å^2^ of surface area is buried in the ReoM dimer interface, representing 9% of the total solvent accessible surface area. The similarity of the monomers and the dimeric assemblies of ReoM and IreB is underlined by the 1.5 and 1.7 Å r.m.s.d. values, respectively, on global secondary structure superposition matching 74 Cα atoms from each protomer in the comparison.

**Figure 6—figure supplement 1.**
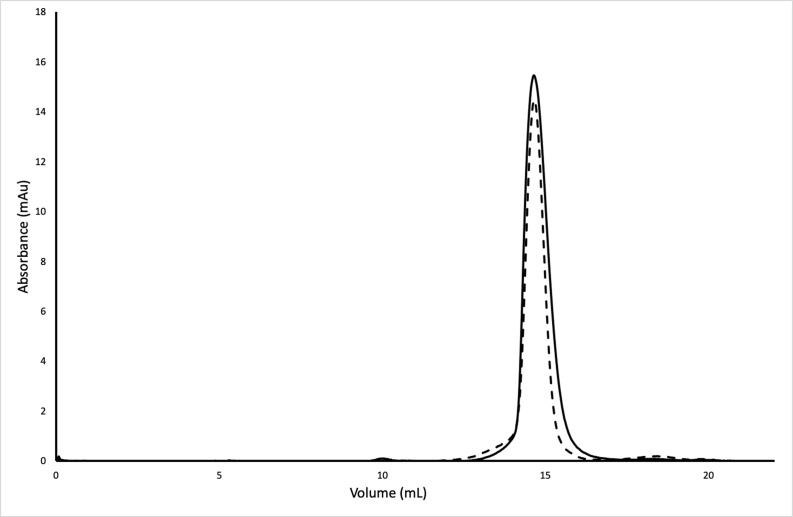
ReoM and P-ReoM have the same oligomeric state. Size exclusion chromatography of ReoM and P-ReoM showed that phosphorylation of ReoM does not alter its oligomeric state. Both ReoM (solid) and P-ReoM (dashed) had an elution volume at 14.6 mL.

**Figure 6—figure supplement 2.**
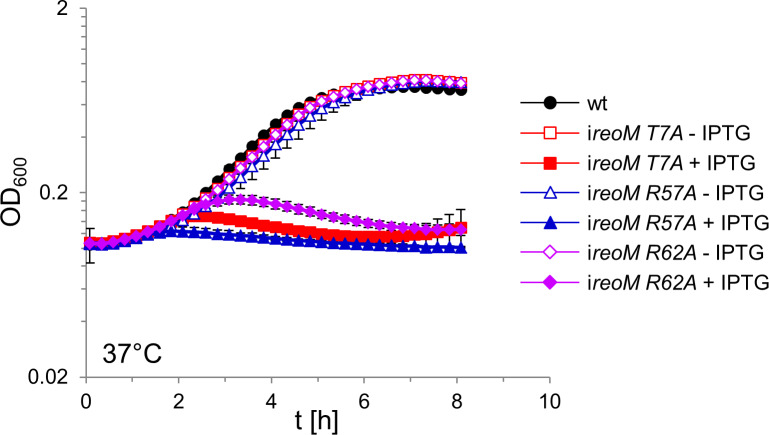
Lethality of ReoM R57A and R62A substitutions. *L. monocytogenes* strains EGD-e (wt), LMSW52 (i*reoM T7A*), LMSW125 (i*reoM R57A*), and LMSW126 (i*reoM R62A*) were grown in BHI broth ±1 mM IPTG at 37°C. The experiment was repeated three times and average values and standard deviations are shown.

**Figure 6—figure supplement 3.**
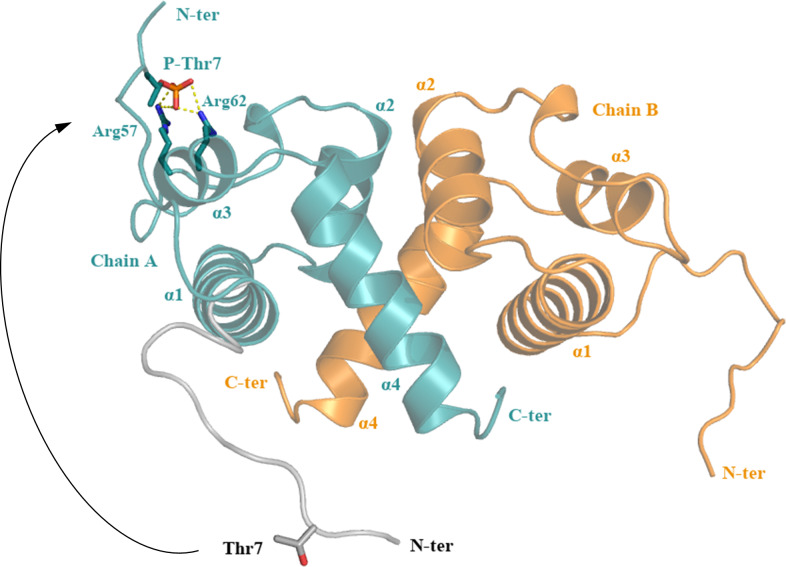
A possible conformational change of the flexible ReoM N-terminus induced by phosphorylation. The N-terminus of ReoM might undergo a substantial movement after phosphorylation that would be stabilised in its new conformation by electrostatic interactions between the negatively charged phosphate of Thr7 ~P and the positively charged Arg57/Arg62 pair.

**Table 1.** Summary of the data collection and refinement statistics for ReoM.

Data collection	
Beamline	Diamond I03
Wavelength (Å)	0.976
Resolution (Å)	74.45–1.60 (1.63–1.60)*
Space group	P 2_1_ 2_1_ 2_1_
*a, b, c* (Å)	38.79, 58.62, 74.45
α, β, γ (°)	90, 90, 90
R_pim_	0.064 (0.533)
CC (1/2) (%)	98.6 (62.0)
<I>/<σ(I)>	8.2 (2.2)
Completeness (%)	99.8 (99.8)
Redundancy	4.8 (4.9)
Total observations	111229 (5581)
Unique reflections	23059 (1129)
Refinement	
R_work_ (%)	15.3
R_free_ (%)	21.4
Solvent content (%)	38.0
# atoms	
Protein	1399
Ligand/ion	20
Water	94
B-factors (Å^2^)	
Protein	26.4
Ligand/ion	50.5
Water	37.7
R.m.s deviations	
Bonds (Å)	0.015
Angles (°)	1.79

^*^Where values in parentheses refer to the highest resolution shell.

Other than the compact helical bundle of ReoM, there is a ~ 16 residue-long N-terminal tail, with B-factors 25% higher than the rest of the protein, prior to the start of α-helix one at residue Ile17. The equivalent N-terminal region is also disordered in the NMR structure of IreB (Hall et al., 2017). Despite the absence of secondary structure, the ReoM model covering this region could be built with confidence from Asp5 in chain A and Asp2 in chain B (Figure 6C). Consequently, it is possible to visualise Thr7, the target for phosphorylation by PrkA, in the flexible N-terminal region in both chains. The side chain of Thr7 in both chains makes no intramolecular interactions and is thus amenable to phosphorylation by PrkA. Despite multiple attempts, however, no crystals of P-ReoM could be grown. Several possible ReoM responses to phosphorylation exist including a change in oligomeric state, as observed quite commonly in response regulators in order to bind more effectively to promoter regions to effect transcription (Johnson and Lewis, 2001). However, analysis of the oligomeric state of P-ReoM by size exclusion chromatography revealed that the protein behaved in solution the same as to unphosphorylated ReoM (Figure 6—figure supplement 1).

Alternatively, the presence of a sulphate ion (a component of the crystallisation reagent) adjacent to the sidechain of Thr7 could mimic what P-ReoM might look like (Figure 6C). The sulphate ion is captured by a positively-charged micro-environment from a symmetry-equivalent molecule. ReoM could thus react to phosphorylation by a substantial movement of Thr7 to interact with this positively-charged surface, which comprises arginines with levels of conservation (Arg57 [57% conserved], Arg62 [99%], Arg66 [76%], Arg70 [98%]) amongst all 2909 ReoM homologues present at NCBI approaching that of Thr7 (96%). We subsequently made alanine substitutions of each of these arginines in *reoM*. Whereas the R66A and R70A mutations were without any effect on growth (data not shown), expression of ReoM R57A and R62A mutations were as lethal as expression of ReoM T7A (Figure 6—figure supplement 2). Thus, Arg57 and Arg62 might co-ordinate P-Thr7, stabilising the conformation and position of the flexible N-terminal region (Figure 6—figure supplement 3), though confirmation of the molecular consequences of ReoM phosphorylation remain to be determined.

### Control of MurA stability and PG biosynthesis by the PrkA/PrpC protein kinase/phosphatase pair

**Figure 7.**
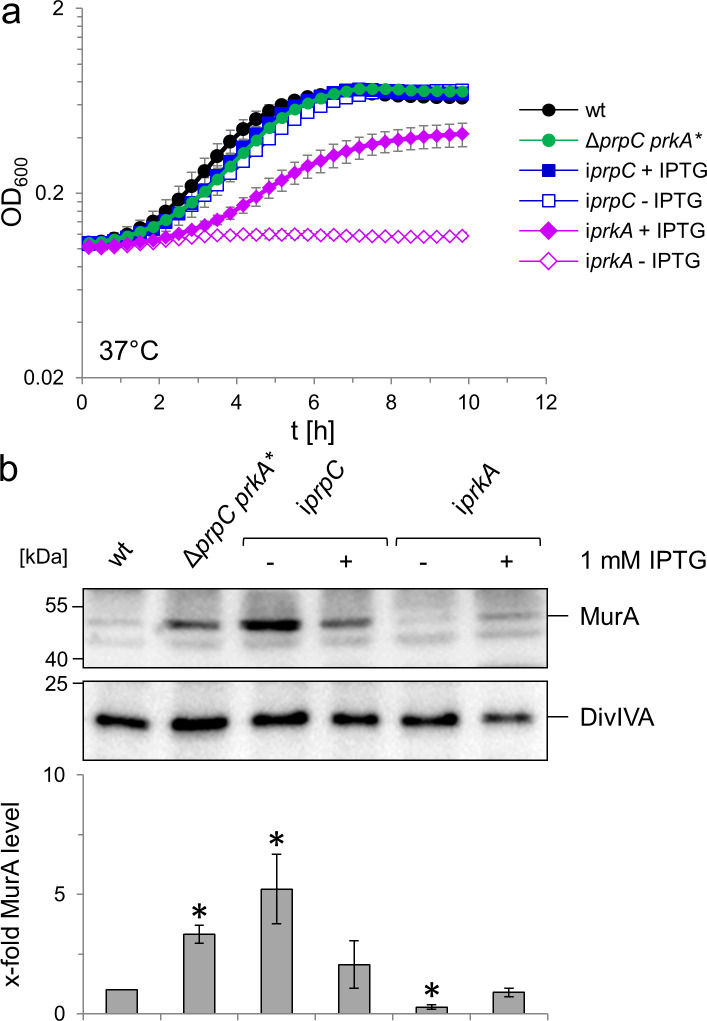
Effect of *prkA* and *prpC* mutations on growth and MurA levels of *L. monocytogenes*. (**A**) Contribution of PrkA and PrpC to *L. monocytogenes* growth. *L. monocytogenes* strains EGD-e (wt), LMSW76 (ΔprpC prkA*), LMSW83 (i*prpC*) and LMSW84 (i*prkA*) were grown in BHI broth ±1 mM IPTG at 37°C in a microtiter plate reader. The experiment was repeated three times and average values and standard deviations are shown. (**B**) Contribution of PrkA and PrpC to MurA stability. Western blots showing cellular levels of MurA (top) and DivIVA (middle) in the same set of strains as in panel A and quantification of MurA signals by densitometry (below). Average values and standard deviations calculated from three independent experiments are shown. Asterisks indicate statistically significant differences (p<0.05, *t*-test).

To study the contribution of the PrkA/PrpC couple to PG biosynthesis in more detail, we aimed to construct *prkA* and *prpC* deletion mutants, but failed to delete *prkA*. However, *prkA* could be deleted in the presence of an IPTG-inducible ectopic *prkA* copy and the resulting strain (LMSW84) required IPTG for growth (Figure 7A), demonstrating the essentiality of this gene. The essentiality of *prkA* in our hands is consistent with results by others who have also shown that *prkA* can only be inactivated in the presence of a second copy (Pensinger et al., 2014). Repeated attempts to delete *prpC* finally yielded a single Δ*prpC* clone (LMSW76). Genomic sequencing of this strain, which grew at a similar rate to wild type (Figure 7A), confirmed the successful deletion of *prpC* but also identified a trinucleotide deletion in the *prkA* gene (designated prkA*), effectively removing the complete codon of Gly18 that is part of a conserved glycine-rich loop important for ATP binding (Rakette et al., 2012). Presumably, this mutation reduces the PrkA kinase activity to balance the absence of PrpC. By contrast, *prpC* could be deleted readily in the presence of a second IPTG-dependent copy of *prpC* and growth of the resulting strain (LMSW83) did not require IPTG, most likely explained by promoter leakiness in the absence of IPTG (Figure 7A). The viability of the i*prpC* mutant shows that the *prpC* deletion had no polar effects on the expression of the downstream *prkA*. That *prkA* and *prpC* are both essential suggests that some of their substrates must be phosphorylated and unphosphorylated, respectively, to be active. Next, the effect of *prkA* and *prpC* mutations on MurA accumulation was analysed by western blotting. Intermediate MurA accumulation was evident in the ΔprpC prkA* strain, while full accumulation of MurA was observed in PrpC-depleted cells. By contrast, no MurA was detected in cells depleted for PrkA (Figure 7B). Therefore, PrkA and PrpC inversely contribute to the accumulation of MurA, suggesting that phosphorylated ReoM favours MurA accumulation, while un-phosphorylated ReoM counteracts this process. In good agreement, depletion of PrkA strongly increased ceftriaxone susceptibility, while inactivation of *prpC* caused increased ceftriaxone resistance (Figure 3A).

### Deletion of *reoM*, *reoY* or *clpC* eliminates *prkA* essentiality

**Figure 8.**
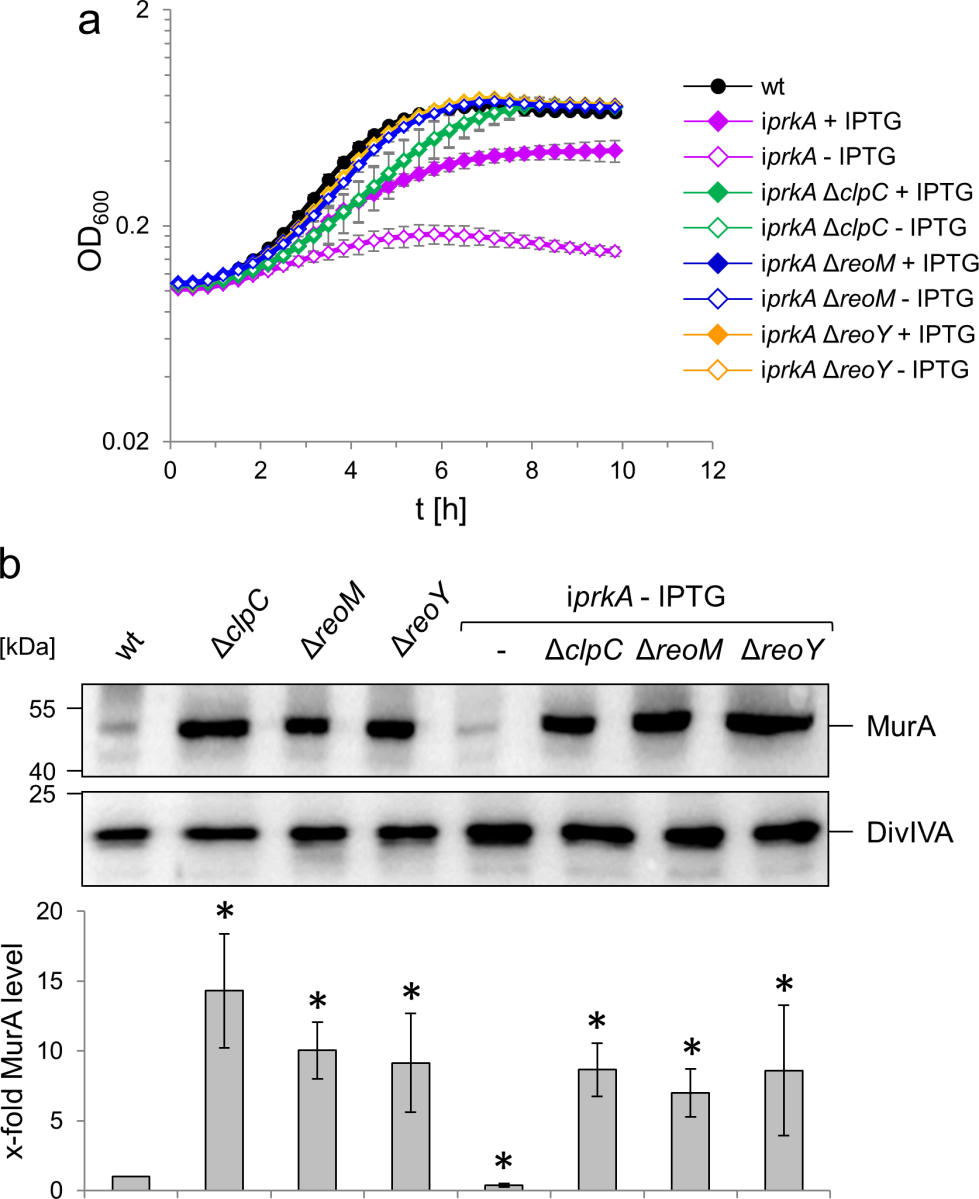
PrkA essentiality depends on *reoM*, *reoY* and *clpC*. (**A**) Effect of *reoM*, *reoY* and *clpC* deletions on *prkA* essentiality. *L. monocytogenes* strains EGD-e (wt), LMSW84 (i*prkA*), LMSW89 (i*prkA* Δ*reoM*), LMSW90 (i*prkA* Δ*reoY*) and LMSW91 (i*prkA* Δ*clpC*) were grown in BHI broth ±1 mM IPTG at 37°C in a microtiter plate reader. The experiment was repeated three times and average values and standard deviations are shown. (**B**) *clpC, reoM* and *reoY* deletions overcome MurA degradation in PrkA-depleted cells. Western blot showing MurA levels in *L. monocytogenes* strains EGD-e (wt), LMJR138 (Δ*clpC*), LMSW30 (Δ*reoM*), LMSW32 (Δ*reoY*), LMSW84 (i*prkA*), LMSW89 (i*prkA* Δ*reoM*), LMSW90 (i*prkA* Δ*reoY*) and LMSW91 (i*prkA* Δ*clpC*, top). A parallel DivIVA western blot was used as loading control (middle). Quantification of MurA signals by densitometry (below). Average values and standard deviations calculated from three independent experiments are shown. Asterisks indicate statistically significant differences (p<0.05, *t*-test).

**Figure 9.**
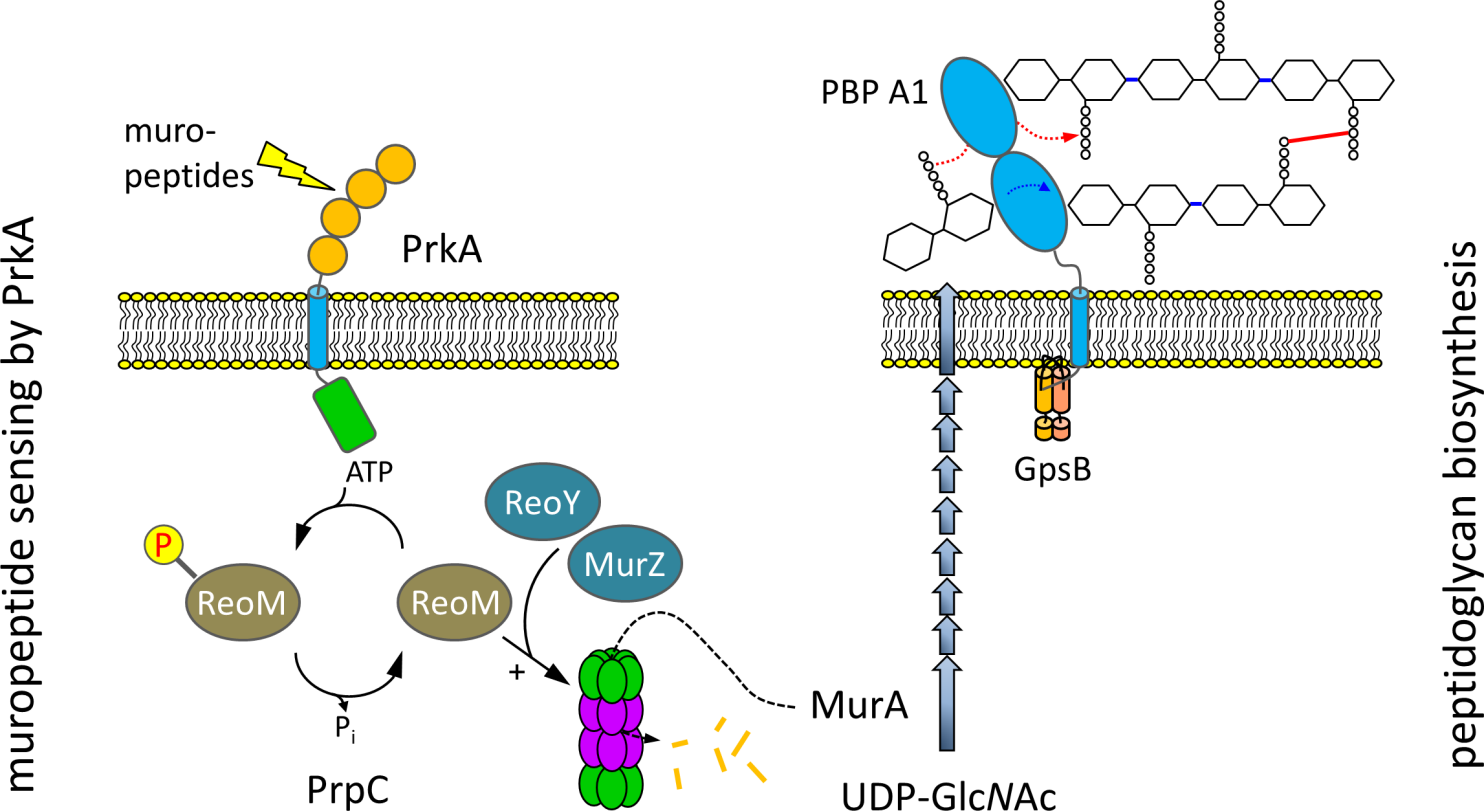
ReoM links PrkA-dependent muropeptide sensing with peptidoglycan biosynthesis. Model illustrating the role of ReoM as substrate of PrkA and as regulator of ClpCP. PrkA recognises free muropeptides, which activate PrkA to phosphorylate ReoM. In its unphosphorlyated form, ReoM is an activator of ClpCP-dependent degradation of MurA, the first enzyme of peptidoglycan biosynthesis, and ReoY and MurZ contribute to this process. By phosphorylating ReoM, PrkA prevents ClpCP-dependent MurA degradation so that MurA accumulates and peptidoglycan biosynthesis can occur. Please note that there is a lesser degree of conservation in the fourth PASTA domain of PrkA.

In order to test whether the essentiality of *prkA* could be explained by stimulated MurA degradation through ClpCP, we first tested the effect of *clpC* on the essentiality of *prkA*. For this purpose, *clpC* was removed from the i*prkA* strain and growth of the resulting strain (LMSW91) was tested. In contrast to the parental i*prkA* strain (LMSW84), which required IPTG for growth, strain LMSW91 was viable without IPTG (Figure 8A) thus confirming that the essentiality of PrkA depends on ClpC. We next wondered whether ReoM and ReoY were also required for PrkA essentiality and consequently deleted their genes from the i*prkA* background to test this. Again, the resulting strains did not require IPTG for growth in contrast to the parental i*prkA* strain (Figure 8A). In good agreement with these findings, deletion of *clpC*, *reoM* or *reoY* all stabilised MurA in PrkA-depleted cells (Figure 8B), showing that the stimulated degradation of MurA that we observe in cells depleted for PrkA (Figure 7B) is dependent on any one of these three proteins. These results together permit a model of genetic interactions to be proposed (Figure 9) that starts with PrkA and its downstream substrate ReoM. ReoY, MurZ and ClpC in turn are positioned downstream of ReoM (as indicated by the experiments shown in Figure 5E) to control MurA stability. To further substantiate this concept, physical interactions between ReoM, ReoY, ClpC, ClpP and MurA were determined in bacterial two hybrid experiments, which revealed that ReoY interacted with ClpC, ClpP and ReoM. In turn, ReoM interacted with MurA (Figure 8—figure supplement 1), which suggests that ReoM and ReoY could bridge the interaction of ClpCP with its substrate MurA.

**Figure 8—figure supplement 1.**
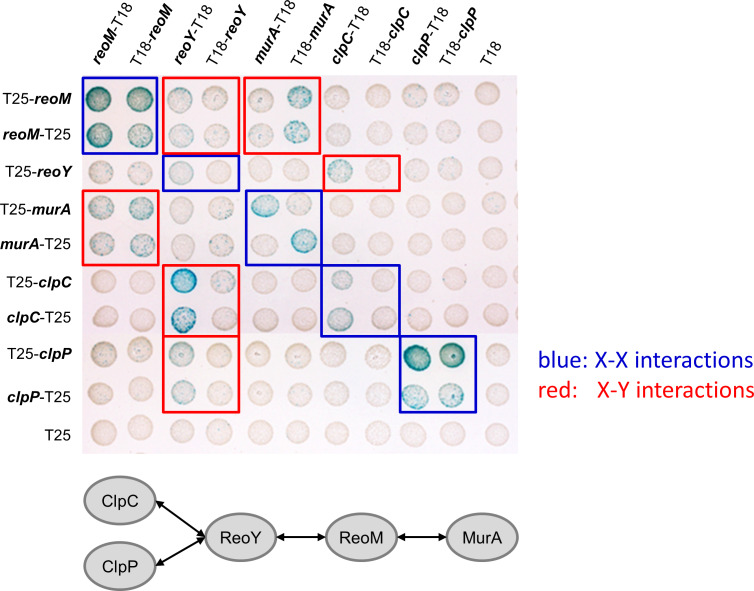
Bacterial two hybrid experiment showing interactions between MurA, ReoM, ReoY, ClpC and ClpP. Plasmids carrying fusions of the T18- and T25-fragments of *Bordetella pertussis* adenylate cyclase fused to MurA, ReoM, ReoY, ClpC and ClpP of *L. monocytogenes* were cotransformed into *E. coli* BTH101 and plated on selective LB agar plates containing X-Gal. Formation of blue colonies indicates interaction between the tested proteins. An illustration summarising all detected protein-protein interactions is shown below. Please note that all *murZ* fusions and the *reoY*-T25 fusion were not functional and were therefore not included.

## Discussion

With ReoM we have identified a missing link in a regulatory pathway that enables Firmicute bacteria to respond to PG biosynthesis fluctuations associated with growth and division. In *L. monocytogenes*, the sensory module of this pathway comprises the membrane integral protein kinase PrkA and the cognate protein phosphatase PrpC, their newly discovered substrate ReoM and the associated factors ReoY and MurZ, which together regulate ClpCP activity, the effector protease that acts on MurA (Figure 9). It has been demonstrated previously that the kinase activity of PrkA homologues was activated by muropeptides (Mir et al., 2011; Shah et al., 2008) or the PG precursor lipid II (Hardt et al., 2017). Muropeptides were released from the cell wall during normal PG turnover, and their release was intensified when PG hydrolysis prevailed over PG biosynthesis (Vollmer et al., 2008b; Irazoki et al., 2019), whereas blocking PG chain elongation by moenomycin treatment caused the accumulation of lipid-linked PG precursors (Kohlrausch and Höltje, 1991). Thus, both types of molecules accumulated when PG biosynthesis was inhibited and could represent useful signals for detecting imbalances in cell wall biosynthesis. Our data are consistent with a model in which PrkA-phosphorylated ReoM no longer activates ClpCP, which leads to MurA stabilisation and the activation of PG biosynthesis (Figure 9). In *B. subtilis*, this effect is supported by stabilisation of GlmS (Figure 2—figure supplement 3A), another ClpCP substrate but which acts in front of MurA as the first enzyme of the UDP-Glc*N*Ac-generating GlmSMU pathway.

How ReoM and ReoY exert their effect on ClpCP is currently unknown, but a fascinating possibility would be a function like to that of an adaptor protein to target protein substrates to ClpCP for degradation. Several ClpC adaptors for different substrates are known in *B. subtilis* (Kirstein et al., 2009; Mulvenna et al., 2019), but an adaptor for *Bs*MurAA is not among them (Kock et al., 2004; Kirstein et al., 2009). Like ReoM, the ClpC adaptor McsB from *B. subtilis* is also subject to phosphorylation, but - unlike ReoM - it targets its substrate CtsR to the ClpCP machinery only when phosphorylated (Kirstein et al., 2007). Either ReoM or ReoY could act as this adaptor, leaving a subsidiary function for the other respective protein. Alternatively, both proteins could work in tandem, where each of them is equally needed for ClpCP-dependent MurA degradation since the phenotypes of *reoM* and *reoY* mutants were identical with respect to MurA stability. However, overexpression or deletion of *reoM* altered the phenotype of the Δ*gpsB* mutant, but that of *reoY* was without phenotype (Figure 1—figure supplement 1, Figure 1—figure supplement 2). ReoY, restricted to the *Bacilli*, also showed a narrower phylogenetic distribution than ReoM, which is found across different *Firmicutes* (Figure 6B). Thus, it seems that ReoM might have a more generalised role, whereas ReoY could play a subordinate function in control of MurA degradation by ClpCP. The role of the MurA homologue MurZ in this process is entirely unclear, but our genetic data now place it downstream of ReoM (Figure 8C). Furthermore, arginine phosphorylation targets proteins to ClpCP for degradation (Trentini et al., 2016). *L. monocytogenes* MurA contains 17 arginines and MurAA of *B. subtilis* has been found in complex with the protein arginine phosphatase YwlE (Elsholz et al., 2012). The possibility that MurA proteins could also require arginine phosphorylation to be accepted as a substrate by ClpCP offers additional control possibilities for ReoM/ReoY/MurZ to modulate MurA levels.

*L. monocytogenes prkA* is essential, but *prkA* homologues in other species are dispensable (Gaidenko et al., 2002; Cuenot et al., 2019; Kristich et al., 2007; Débarbouillé et al., 2009; Nováková et al., 2005). At least in some of them (such as *E. faecalis*, *S. aureus* and *S. pneumoniae*), the primary MurA enzyme can be functionally replaced by a second paralogue (Blake et al., 2009; Vesić and Kristich, 2012; Du et al., 2000), so that proteolytic degradation of the primary enzyme can be tolerated. By contrast, *prkC* is dispensable to *Clostridioides difficile* despite encoding only one copy of the essential MurA gene (Cuenot et al., 2019; Sapkota et al., 2020); *B. subtilis prkC* can also be deleted even though the primary MurA enzyme cannot be replaced by the secondary one (Kock et al., 2004; Gaidenko et al., 2002). Probably, control of MurA degradation by ClpCP is somewhat relaxed in these latter two species.

A screen for *gpsB* suppressors in *S. pneumoniae* did not yield *reoM* mutations (and these strains do not contain *reoY*, consistent with a subordinate function for this gene), but instead suppressor mutations were found that affect *phpP*, which encodes a Ser/Thr protein phosphatase that acts in concert with StkP, the PASTA-eSTK of this organism (Rued et al., 2017; Lewis, 2017). Absence or inactivation of PhpP triggered an increase in StkP-dependent protein phosphorylation levels in the pneumococcus (Rued et al., 2017; Ulrych et al., 2016). It is tempting to speculate that loss of PhpP activity in this suppressor also triggers P-ReoM formation that, according to our model, would help to stabilise MurA and thus suppress the Δ*gpsB* phenotype. Interestingly, another *S. pneumoniae gpsB* suppressor was identified that carries a duplication of a ~ 150 kb genomic fragment (Rued et al., 2017), a region that includes the open reading frame for MurA. Suppression of the *gpsB* phenotype in this instance could also work via MurA accumulation, but this time due to a gene dosage effect.

It is becoming increasingly evident that control of PG biosynthesis in response to cell wall derived signals, via PASTA-eSTKs, is a regulatory capacity common to *Firmicutes* and *Actinobacteria*. CwlM is the critical kinase substrate in the actinobacterium *M. tuberculosis* that, when phosphorylated by PknB, binds to and activates MurA (Boutte et al., 2016). Homologues of CwlM are not present in *L. monocytogenes* or *B. subtilis* and instead these bacteria adjust their MurA levels by controlling MurA turnover in response to PrkA-dependent phosphorylation of ReoM. Consequently, both mechanisms activate PG biosynthesis in a PrkA-dependent manner either by activation or stabilisation of MurA. Presumably *B. subtilis*, and other endospore forming bacteria, re-start PG biosynthesis at the onset of germination in a similar way. Germination of *B. subtilis* spores can be triggered by muropeptides in a manner that depends upon PrkC (Shah et al., 2008), the PASTA-eSTK of *B. subtilis* (Madec et al., 2002). Even though *Bs*PrkC phosphorylates multiple substrates (Ravikumar et al., 2014), whose individual contribution to germination is not known precisely, phosphorylation of ReoM (*aka* YrzL) could be required to restart PG biosynthesis in germinating *B. subtilis* cells by stabilising MurAA. Moreover, an *E. faecalis* mutant lacking the PASTA-eSTK IreK was more susceptible to ceftriaxone but overexpression of *Ef*MurAA overcame this defect (Vesić and Kristich, 2012). This implies the possibility that unphosphorylated IreB together with the ReoY homologue of this organism, OG1RF_11272 (Banla et al., 2018), might stimulate MurAA proteolysis in *E. faecalis* as well. Taken together it seems that observations made in different *Firmicutes* are in good agreement with the PrkA→ReoM/ReoY→ClpC→MurA signaling sequence that we propose. The open questions that remain on the molecular mechanism of ClpCP control by ReoM and ReoY will be addressed by future experiments.

## Materials and methods

### Bacterial strains and growth conditions

Table 2 lists all strains used in this study (also see Supplementary file 1). Strains of *L. monocytogenes* were cultivated in BHI broth or on BHI agar plates. *B. subtilis* strains were grown in LB broth at 37°C. Antibiotics and supplements were added when required at the following concentrations: erythromycin (5 µg/ml), kanamycin (50 µg/ml), X-Gal (100 µg/ml) and IPTG (as indicated). *Escherichia coli* TOP10 was used as host for all cloning procedures (Sambrook et al., 1989). Minimal inhibitory concentrations against ceftriaxone were determined as described previously (Rismondo et al., 2015) using E-test strips with a ceftriaxone concentration range of 0.016–256 µg/ml.

**Table 2.** Plasmids and strains used in this study.

Name	Relevant characteristics	Source*/reference
Plasmids		
pIMK3	P*_help_-lacO lacI neo*	Monk et al., 2008
pMAD	*bla erm bgaB*	Arnaud et al., 2004
pUT18	*bla* P*_lac_-cya(T18)*	Karimova et al., 1998
pUT18C	*bla* P*_lac_-cya(T18)*	Karimova et al., 1998
pKT25	*kan* P*_lac_-cya(T25)*	Karimova et al., 1998
p25-N	*kan* P*_lac_-cya(T25)*	Claessen et al., 2008
pJR127	*bla erm bgaB* Δ*clpC (lmo0232)*	Rismondo et al., 2017
pSH246	*bla erm bgaB* Δ*gpsB (lmo1888)*	Rismondo et al., 2016
pJR68	*bla erm bgaB* Δ*murZ (lmo2552)*	Rismondo et al., 2017
pJR71	P*_help_-lacO-murZ lacI neo*	Rismondo et al., 2017
pJR65	P*_help_-lacO-reoM lacI neo*	this work
pJR70	P*_help_-lacO-reoY lacI neo*	this work
pJR83	*bla erm bgaB* ∆*reoY (lmo1921)*	this work
pJR101	*kan* P*_lac_-cya(T25)-reoM*	this work
pJR102	*kan* P*_lac_-reoM-cya(T25)*	this work
pJR103	*bla* P*_lac_-reoM-cya(T18)*	this work
pJR104	*bla* P*_lac_-cya(T18)-reoM*	this work
pJR109	*kan* P*_lac_-cya(T25)-reoY*	this work
pJR111	*bla* P*_lac_-cya(T18)-reoY*	this work
pJR116	*kan* P*_lac_-cya(T25)-murA*	this work
pJR117	*kan* P*_lac_-murA-cya(T25)*	this work
pJR118	*bla* P*_lac_-murA-cya(T18)*	this work
pJR119	*bla* P*_lac_-cya(T18)-murA*	this work
pJR121	*bla* P*_lac_-reoY-cya(T18)*	this work
pJR126	*bla erm bgaB* ∆*reoM (lmo1503)*	this work
pSW29	P*_help_-lacO-reoM T7A lacI neo*	this work
pSW30	P*_help_-lacO-reoM T7D lacI neo*	this work
pSW36	*bla erm bgaB* Δ*prkA (lmo1820)*	this work
pSW37	*bla erm bgaB* Δ*prpC (lmo1821)*	this work
pSW38	P*_help_-lacO-prkA lacI neo*	this work
pSW39	P*_help_-lacO-prpC lacI neo*	this work
pSW43	*kan* P*_lac_-cya(T25)-clpC*	this work
pSW44	*kan* P*_lac_-cya(T25)-clpP*	this work
pSW45	*kan* P*_lac_- clpC-cya(T25)*	this work
pSW46	*kan* P*_lac_-clpP-cya(T25)*	this work
pSW47	*bla* P*_lac_-clpC-cya(T18)*	this work
pSW48	*bla* P*_lac_-clpP-cya(T18)*	this work
pSW49	*bla* P*_lac_-cya(T18)-clpC*	this work
pSW50	*bla* P*_lac_-cya(T18)-clpP*	this work
pSW55	P*_help_-lacO-reoM R66A lacI neo*	this work
pSW56	P*_help_-lacO-reoM R70A lacI neo*	this work
pSW58	P*_help_-lacO-reoM R57A lacI neo*	this work
pSW59	P*_help_-lacO-reoM R62A lacI neo*	this work
*B. subtilis strains*		
168	wild type, lab collection	
BKE00860	Δ*clpC*	Koo et al., 2017
BKE22180	Δ*gpsB*	Koo et al., 2017
BKE22580	Δ*ypiB (reoY)*	Koo et al., 2017
BKE27400	Δ*yrzL (reoM)*	Koo et al., 2017
*L. monocytogenes* strains		
EGD-e	wild-type, serovar 1/2a strain	Glaser et al., 2001
LMJR19	Δ*gpsB (lmo1888)*	Rismondo et al., 2016
LMJR104	∆*murZ (lmo2552)*	Rismondo et al., 2017
LMJR116	*attB::*P*_help_-lacO-murA lacI neo*	Rismondo et al., 2017
LMJR123	Δ*murA (lmo2526) attB::*P*_help_-lacO-murA lacI neo*	Rismondo et al., 2017
LMJR138	Δ*clpC (lmo0232)*	Rismondo et al., 2017
*shg8*	Δ*gpsB reoY* H87Y	this work
*shg10*	Δ*gpsB reoY* TAA74	this work
*shg12*	Δ*gpsB reoM* RBS mutation	this work
LMJR96	∆*gpsB attB::*P*_help_-lacO-reoM lacI neo*	pJR65 → LMJR19
LMJR102	*attB::*P*_help_-lacO-reoM lacI neo*	pJR65 → EGD-e
LMJR106	∆*gpsB attB::*P*_help_-lacO-reoY lacI neo*	pJR70 → LMJR19
LMJR120	Δ*gpsB* Δ*reoY*	pJR83 ↔ LMJR19
LMJR137	Δ*gpsB* Δ*reoM*	pJR126 ↔ LMJR19
LMJR171	Δ*clpC* Δ*murZ*	pJR127 ↔ LMJR104
LMSW30	Δ*reoM (lmo1503)*	pJR126 ↔ EGD-e
LMSW32	Δ*reoY (lmo1921)*	pJR83 ↔ EGD-e
LMSW50	Δ*clpC* Δ*reoM*	pJR127 ↔ LMSW30
LMSW51	Δ*clpC* Δ*reoY*	pJR127 ↔ LMSW32
LMSW52	Δ*reoM attB::*P*_help_-lacO-reoM T7A lacI neo*	pSW29 → LMSW30
LMSW53	Δ*reoM attB::*P*_help_-lacO-reoM T7D lacI neo*	pSW30 → LMSW30
LMSW57	Δ*reoM attB::*P*_help_-lacO-reoM lacI neo*	pJR65 → LMSW30
LMSW72	Δ*reoM attB::*P*_help_-lacO-reoM T7A lacI neo* Δ*clpC*	pJR127 ↔ LMSW52
LMSW76	ΔprpC prkA^*^	pSW37 ↔ EGD-e
LMSW80	*attB::*P*_help_-lacO-prkA lacI neo*	pSW38 → EGD-e
LMSW81	*attB::*P*_help_-lacO-prpC lacI neo*	pSW39 → EGD-e
LMSW83	Δ*prpC attB::*P*_help_-lacO-prpC lacI neo*	pSW37 ↔ LMSW81
LMSW84	Δ*prkA attB::*P*_help_-lacO-prkA lacI neo*	pSW36 ↔ LMSW80
LMSW89	Δ*prkA attB::*P*_help_-lacO-prkA lacI neo* Δ*reoM*	pJR126 ↔ LMSW84
LMSW90	Δ*prkA attB::*P*_help_-lacO-prkA lacI neo* Δ*reoY*	pJR83 ↔ LMSW84
LMSW91	Δ*prkA attB::*P*_help_-lacO-prkA lacI neo* Δ*clpC*	pJR127 ↔ LMSW84
LMSW117	Δ*reoM* Δ*reoY*	pJR126 ↔ LMSW32
LMSW118	Δ*reoY* Δ*murZ*	pJR68 ↔ LMSW32
LMSW119	Δ*reoM* Δ*murZ*	pJR68 ↔ LMSW30
LMSW120	Δ*reoM attB::*P*_help_-lacO-reoM R66A lacI neo*	pSW55 → LMSW30
LMSW121	Δ*reoM attB::*P*_help_-lacO-reoM R70A lacI neo*	pSW56 → LMSW30
LMSW123	Δ*reoM attB::*P*_help_-lacO-reoM T7A lacI neo* Δ*reoY*	pSW29 → LMSW117
LMSW124	Δ*reoM attB::*P*_help_-lacO-reoM T7A lacI neo* Δ*murZ*	pSW29 → LMSW119
LMSW125	Δ*reoM attB::*P*_help_-lacO-reoM R57A lacI neo*	pSW58 → LMSW30
LMSW126	Δ*reoM attB::*P*_help_-lacO-reoM R62A lacI neo*	pSW59 → LMSW30
LMSW138	Δ*reoY attB::*P*_help_-lacO-reoY lacI neo*	pJR70 → LMSW32
LMSW139	Δ*murZ attB::*P*_help_-lacO-murZ lacI neo*	pJR71 → LMJR104

^*^The arrow (→) stands for a transformation event and the double arrow (↔) indicates gene deletions obtained by chromosomal insertion and subsequent excision of pMAD plasmid derivatives (see experimental procedures for details).

### General methods, manipulation of DNA and oligonucleotide primers

All key resources used in this study are listed in Supplementary file 1. Standard methods were used for transformation of *E. coli* and isolation of plasmid DNA (Sambrook et al., 1989). Transformation of *L. monocytogenes* was carried out as described by others (Monk et al., 2008). Restriction and ligation of DNA was performed according to the manufacturer´s instructions. All primer sequences are listed in Table 3 (also see Supplementary file 1).

**Table 3.** Oligonucleotides used in this study.

Name	Sequence (5´→3´)
JR163	GCGCCCATGGCTAAGGCATCCATTTCAATAGACGAGAAG
JR164	GCGCGTCGACTTATTCTTTTTCCGTATCCATTTGCTGTA
JR169	GCGCCCATGGATTCAAAAGATCAAACAATGTTTTACAACTTC
JR170	GCGCGTCGACTCATTTCTCACCAATTTCGTTATTTTTCAG
JR197	GCGCGGATCCCAATTATTTCGAATGGTGCGGTGTC
JR198	TCCTTATTCGTCGACCATCTTTCCTCAGTCCCTTCCTG
JR199	GGAAAGATGGTCGACGAATAAGGAATAAATCCTAGTTAGTAGGG
JR200	CGCGCGAATTCCCAAGACTCAACCTCTTTCACTC
JR249	GCGCCTGCAGAAAAAATTATTGTACGCGGTGGAAAAC
JR250	GCGCGGTACCGCGAATAAAGACGCTAAGTTTGTTACATCG
JR253	GCGCTCTAGAAAAGGCATCCATTTCAATAGACGAG
JR254	GCGCGGTACCTCTTTTTCCGTATCCATTTGCTG
JR255	GCGCTCTAGATTCAAAAGATCAAACAATGTTTTACAAC
JR256	GCGCGGTACCTTCTCACCAATTTCGTTATTTTTCAG
JR257	GCGCCTGCAGGGAAAAAATTATTGTACGCGGTGGAAAAC
JR264	GCGCAGATCTGGCAAATACAGCATTGAACTATGTG
JR265	GCGCGGATCCAATCGAAGCACCTCATTCCTTC
JR266	GCGCGGATCCATGAGAATAATGGGTTTAGATGTCGGC
JR267	GCGCGTCGACGCTAGGAATGTAGCAAGGATTTCTTC
SHW815	GATCTATCGATGCATGCCATGGGCTAAATGACCAAGGAATTACCG
SHW816	CGCGTCGGGCGATATCGGATCCTTTCTTCCGCGTTTTGGTAACG
SHW817	CAATCATCATTTTAAAAGCACCTCACTATTTTTCAG
SHW818	TGCTTTTAAAATGATGATTGGTAAGCGATTAAGC
SHW819	GATCTATCGATGCATGCCATGGAGATAGAGGCAGAATAAGACATC
SHW820	CGCGTCGGGCGATATCGGATCCGGTATTTACAACCACTACGTCG
SHW821	CGTTCTTATTTCATGAAGCATCCCTCCCTTTC
SHW822	TGCTTCATGAAATAAGAACGGAGGAAATGTGCTG
SHW830	GCGCGCTCTAGATGGACGATTTACGCAAAGAGCTCAG
SHW831	GCGCGCGGTACCTTAGCTTTTACTTTTTTAGAGGTTGTTTTC
SHW832	GCGCGCTCTAGAAATTCCAACAGTAATTGAACAAACTAGC
SHW833	GCGCGCGGTACCCCTTTTAAGCCAGATTTATTAATGATAATATC
SW77	GTAAAACATTGCTTGATCTTTTGAATCCATGGGTTTCAC
SW78	GATCAAGCAATGTTTTACAACTTCGGCGATGATTC
SW79	GTAAAACATGTCTTGATCTTTTGAATCCATGGGTTTCAC
SW80	GATCAAGACATGTTTTACAACTTCGGCG ATGATTC
SW110	GCGCGCGGATCCATGCATGCAGAATTTAGAACAGATAG
SW111	GCGCGCGTCGACTCATGAAGCATCCCTCCCTTTC
SW112	GCGCGCGGATCCATGATGATTGGTAAGCGATTAAGCG
SW113	GCGCGCGTCGACTTAATTTGGATAAGGGACTGTACCTTC
SW136	CTAAACGAGCTATCATACTTCTAGCATCCTTGTGAC
SW137	GTATGATAGCTCGTTTAGAACGAGATGAAATTATCGAG
SW138	AATTTCATCTGCTTCTAAACGACGTATCATACTTCTAGC
SW139	GTTTAGAAGCAGATGAAATTATCGAGGAACTTGTCAAAG
SW144	CCTTGTGAGCAGGAATATAAGCAGGATCGCCTG
SW145	TATATTCCTGCTCACAAGGATGCTAGAAGTATGATAC
SW146	GTATCATACTTGCAGCATCCTTGTGACGAGGAATATAAG
SW147	GGATGCTGCAAGTATGATACGTCGTTTAGAACGAG
Lmo1503F	GCTATACCATGGATTCAAAAGATCAAACAATGTTTTACAAC
Lmo1503R	CGATATCTCGAGTCATTTCTCACCAATTTCGTTATTTTTCAG
PrkAF	GCTATACCATGGCAATGATGATTGGTAAGCGATTAAGCG
PrkAR	CGATATCTCGAGTCATTTTTTCTTTTTCTTATCTTTTTTCTCCTCAGG
PrpCF	GCTATACCATGGCAATGCATGCAGAATTTAGAACAGATAGAG
PrpCR	CGATATCTCGAGTCATGAAGCATCCCTCCCTTTC

### Construction of plasmids for recombinant protein expression

The plasmids for expressing recombinant versions of ReoM, PrkA-KD and PrpC were prepared by first amplifying the corresponding genes (*reoM*, *lmo1820* and *lmo1821*) from *L. monocytogenes* EGD-e genomic DNA using primer pairs Lmo1503F/Lmo1503R, PrkAF/PrkAR, and PrpCF/PrpCR, respectively. The PCR products were individually ligated between the NcoI and XhoI sites of pETM11 (Peränen et al., 1996). All mutagenesis was carried out using the Quikchange protocol and the correct sequence of each plasmid and mutant constructed was verified by Sanger DNA sequencing (Eurofins Genomics).

### Construction of plasmids for generation of *L. monocytogenes* strains

Plasmid pJR65 was constructed for the inducible expression of *reoM*. To this end, the *reoM* open reading frame was amplified by PCR using the oligonucleotides JR169/JR170 and cloned into pIMK3 using NcoI/SalI. The T7A and T7D mutations were introduced into *reoM* of plasmid pJR65 by quickchange mutagenesis using the primer pair SW77/SW78 and SW79/SW80, respectively. The R57A, R62A R66A and R70A, mutations were introduced into pJR65 in the same way, but using primer pairs SW144/SW145, SW146/SW147, SW136/SW137 and SW138/SW139, respectively.

Plasmid pJR70 was constructed for inducible *reoY* expression. For this purpose, *reoY* was amplified using the primer pair JR163/JR164 and cloned into pIMK3 using NcoI/SalI.

Plasmid pSW38, for IPTG-inducible *prkA* expression, was constructed by amplification of *prkA* using the oligonucleotides SW112/SW113 and the subsequent cloning of the generated fragment into pIMK3 using BamHI/SalI. Plasmid pSW39, for IPTG-controlled expression of *prpC*, was constructed analogously, but using the oligonucleotides SW110/SW111 as the primers.

For construction of plasmid pJR83, facilitating deletion of *reoY*, fragments encompassing ~800 bp up- and down-stream of *reoY* were amplified by PCR with the primer pairs JR197/JR198 and JR199/JR200. Both fragments were spliced together by splicing by overlapping extension (SOE) PCR and cloned into pMAD using BamHI/EcoRI.

Plasmid pJR126 was generated for deletion of *reoM*. Fragments up- and down-stream of *reoM* were PCR amplified using the primers JR264/JR265 and JR266/JR267, respectively. Both fragments were cut with BamHI, fused together by ligation and the desired fragment was amplified from the ligation mixture by PCR using the primers JR264/JR267 and then cloned into pMAD using BglII/SalI.

Plasmid pSW36 was constructed to delete the *prkA* gene. Fragments up- and down-stream of *prkA* were amplified in separate PCRs using the primer pairs SHW819/SHW821 and SHW820/SHW822, respectively. Both fragments were fused together by SOE-PCR and inserted into pMAD by restriction free cloning (van den Ent and Löwe, 2006). Plasmid pSW37, facilitating deletion of *prpC*, was constructed in a similar manner. Up- and down-stream fragments of *prpC* were amplified using the primer pairs SHW815/SHW817 and SHW816/SHW818 and fused together by SOE-PCR. The resulting fragment was inserted into pMAD by restriction free cloning.

Derivatives of pIMK3 were introduced into *L. monocytogenes* strains by electroporation and clones were selected on BHI agar plates containing kanamycin. Plasmid insertion at the *attB* site of the tRNA^Arg^ locus was verified by PCR. Plasmid derivatives of pMAD were transformed into the respective *L. monocytogenes* recipient strains and genes were deleted as described elsewhere (Arnaud et al., 2004). All gene deletions were confirmed by PCR.

### Construction of bacterial two hybrid plasmids

The *reoM* (JR255/JR256), *reoY* (JR253/JR254), *clpC* (SHW830/831) and *clpP* (SHW832/833) genes were amplified using the primer pairs given in brackets and cloned into pUT18, pUT18C, pKT25 and p25-N plasmids using XbaI/KpnI. The *murA* gene was amplified using the oligonucleotides JR249/JR250 for cloning into pKT25 and p25-N using PstI/KpnI or using the JR257/JR250 primer pair for cloning into pUT18 and pUT18C using the same restriction enzymes.

### Bacterial two hybrid experiments

Plasmids carrying genes fused to T18- or the T25-fragments of the *Bordetella pertussis* adenylate cyclase were co-transformed into *E. coli* BTH101 (Karimova et al., 1998) and transformants were selected on LB agar plates containing ampicillin (100 µg ml^−1^), kanamycin (50 µg ml^−1^), X-Gal (0.004%) and IPTG (0.1 mM). Agar plates were photographed after 48 hr of incubation at 30°C.

### Genome sequencing

A total of 1 ng of genomic DNA was used for library generation by the Nextera XT DNA Library Prep Kit according to the manufacturer’s recommendations (Illumina). Sequencing was carried out on a MiSeq benchtop sequencer and performed in paired-end modes (2 × 300 bp) using a MiSeq Reagent Kit v3 cartridge (600-cycle kit). Sequencing reads were mapped to the reference genome *L. monocytogenes* EGD-e (NC_003210.1) (Glaser et al., 2001) by utilising the Geneious software (Biomatters Ltd.). Variants, representing putative suppressor mutations, were identified using the Geneious SNP finder tool. Genome sequences of *shg8*, *shg10*, *shg12* and LMSW76 were deposited at ENA under study number PRJEB35110 and sample accession numbers ERS3927571 (SAMEA6127277), ERS3927572 (SAMEA6127278), ERS3927573 (SAMEA6127279), and ERS3967687 (SAMEA6167687) respectively.

### Isolation of cellular proteins and western blotting

20 ml cells were harvested by centrifugation, washed with ZAP buffer (10 mM Tris.HCl pH7.5, 200 mM NaCl), resuspended in 1 ml ZAP buffer also containing 1 mM PMSF and disrupted by sonication. Centrifugation was used to remove cellular debris and the supernatant was used as total cellular protein extract. Sample aliquots were separated by standard SDS polyacrylamide gel electrophoresis. Gels were transferred onto positively charged polyvinylidene fluoride membranes by semi-dry transfer. ClpC, DivIVA, GlmS, IlvB and MurA were immune-stained using a polyclonal rabbit antiserum recognising *B. subtilis* ClpC (Gerth et al., 2004), DivIVA (Marston et al., 1998), GlmS, IlvB (Gerth et al., 2008) and MurAA (Kock et al., 2004) as the primary antibody and an anti-rabbit immunoglobulin G conjugated to horseradish peroxidase as the secondary one. The ECL chemiluminescence detection system (Thermo Scientific) was used for detection of the peroxidase conjugates on the PVDF membrane in a chemiluminescence imager (Vilber Lourmat). For depletion of PrkA, PrkA depletion strains were grown overnight in the presence of 1 mM IPTG and then again inoculated in BHI broth containing 1 mM IPTG to an OD_600_ = 0.05x00A0 and grown for 3 hr at 37°C. Subsequently, cells were centrifuged, washed and reinoculated in BHI broth without IPTG at the same OD_600_ as before centrifugation. Finally, cells were harvested after 3.5 more hours of growth at 37°C and cellular proteins were isolated.

### Microscopy

Cytoplasmic membranes of exponentially growing bacteria were stained through addition of 1 µl of nile red solution (100 µg ml^−1^ in DMSO) to 100 µl of culture. Images were taken with a Nikon Eclipse Ti microscope coupled to a Nikon DS-MBWc CCD camera and processed using the NIS elements AR software package (Nikon) or ImageJ. Ultrathin section transmission electron microscopy and scanning electron microscopy were performed essentially as described earlier (Rismondo et al., 2015).

### Recombinant protein purification

All proteins were expressed in *E. coli* BL21 (DE3) cells. Cell cultures were grown at 37°C in LB liquid media supplemented with 50 µg mL^−1^ kanamycin to an OD_600_ of 0.6–0.8 before expression was induced by the addition of IPTG to a final concentration of 0.4 mM IPTG. The cultures were incubated at 20°C overnight before the cells from 2 L of cell culture were harvested by centrifugation at 3500 x g for 30 min. The cell pellets were resuspended in 70 mL of buffer A (50 mM Tris.HCl, pH 8, 300 mM NaCl, 10 mM imidazole) with 500 Kunitz units of DNase I and 1 mL Roche complete protease inhibitor cocktail at 25x working concentration. The cells were lysed by sonication, centrifuged at 19000 x g for 20 min and the supernatant was filtered using a 0.45 µm filter. The clarified cell lysate was loaded onto a 5 mL Ni-NTA superflow cartridge (Qiagen), washed with buffer A, and bound proteins were eluted with 50 mM Tris.HCl, pH 8, 300 mM NaCl, 250 mM imidazole. The His_6_-tag of PrkA-KD was cleaved with His-tagged TEV protease (1 mg TEV for 20 mg of protein) at 4°C during an overnight dialysis against a buffer of 50 mM Tris.HCl, pH 8, 300 mM NaCl, 10 mM imidazole, 1 mM DTT; TEV cleavage of ReoM was conducted as above except the dialysis was carried out at 20°C. The proteolysis reaction products were then passed over a 5 mL Ni-NTA superflow cartridge (Qiagen) to remove TEV and uncleaved protein. The proteins that did not bind to the Ni-NTA column were concentrated and loaded onto either a Superdex 75 XK16/60 (GE Healthcare) (ReoM) or a Superdex 200 XK16/60 (GE Healthcare) (PrkA-KD and PrpC) equilibrated with 10 mM Na-HEPES, pH 8, 100 mM NaCl for size exclusion chromatography. Fractions from the gel filtration were analysed for purity by SDS-PAGE, concentrated to 20–40 mg mL^−1^, and small aliquots were snap-frozen in liquid nitrogen for storage at −80°C.

### X-ray crystallography and ReoM structure determination

For ReoM, 23 mg mL^−1^ of protein in 10 mM Na-HEPES pH 8, 100 mM NaCl was subjected to crystallisation by sparse matrix screening using a panel of commercial crystallisation screens. 100 and 200 nL drops of protein and 100 nL of screen solution were dispensed into 96 well MRC crystallisation plates (Molecular Dimensions) by a Mosquito (TTP Labtech) liquid handling robot and the crystallisation plates were stored at a constant temperature of 20°C. The crystals that grew and were subsequently used for diffraction experiments were formed in 0.1 M phosphate/citrate pH 4.2, 0.2 M lithium sulfate, 20 % w/v PEG 1000 from the JCSG + screen and were mounted onto rayon loops directly from the crystallisation drops and cryo-cooled in liquid nitrogen.

Diffraction data were collected on beamline I03 at the Diamond Light Source (DLS) synchrotron. Diffraction images were integrated in MOSFLM (Battye et al., 2011) and scaled and merged with AIMLESS (Evans and Murshudov, 2013). The initial model was generated by molecular replacement in PHASER (McCoy et al., 2007) using the dimeric, 20-conformer ensemble model (PDBid 5US5) of IreB solved by nuclear magnetic resonance (Hall et al., 2017) as a search model. The final model was produced by iterative cycles of model building in COOT (Emsley et al., 2010) with refinement in REFMAC (Murshudov et al., 1997) until convergence. The diffraction data collection and model refinement statistics are summarised in Table 1.

### Protein phosphorylation and dephosphorylation

The effect of phosphorylation and dephosphorylation on ReoM and PrkA-KD proteins was analysed by 20% non-denaturing PAGE. Phosphorylation reactions consisted of 18.5 µM ReoM, 3.7 µM PrkA-KD, 5 mM ATP and 5 mM MgCl_2_, diluted in 10 mM HEPES.HCl pH 8.0 and 100 mM NaCl. Dephosphorylation reactions consisted of 37 µM P-ReoM, 3.7 µM PrkA-KD, 18.5 µM PrpC and 1 mM MnCl_2_, diluted in 10 mM HEPES.HCl pH 8.0 and 100 mM NaCl. In each case controls were loaded at the same concentrations. The reactions were incubated at 37°C for 20 min prior to electrophoresis at 200 V for 2.5 hr on ice.

### Isolation of phosphorylated ReoM

Phosphorylation reactions consisted of 37 µM ReoM, 3.7 µM PrkA-KD, 5 mM ATP and 5 mM MgCl_2_, diluted in 10 mM HEPES.HCl pH 8.0 and 100 mM NaCl, to a total volume of 5 mL. The protein mix was loaded onto a PD 10 desalting column to remove excess ATP and protein fractions were loaded onto a MonoQ 5/50 GL column. Buffer A consisted of 10 mM HEPES.HCl pH 8.0 and 100 mM NaCl and buffer B was 10 mM HEPES.HCl pH 8.0 and 1M NaCl. Bound proteins were eluted over 25 mL with a 15–35% gradient of buffer B.

### Liquid chromatography-mass spectrometry

All liquid chromatography-mass spectrometry (LC-MS) analyses were performed using an Agilent 6530 Q-TOF instrument with electrospray ionisation (ESI) in positive ion mode, coupled to an Agilent 1260 Infinity II LC system, utilising mobile phase of 0.1% (v/v) formic acid in LC-MS grade water (A) and acetonitrile (B). Prior to peptide mapping, 10 μL of purified proteins (~1 mg/ml) were digested using Smart Digest Soluble Trypsin Kit (Thermo Fisher Scientific) according to the manufacturer’s guidelines. Tryptic peptides and intact protein samples were extracted using HyperSep Spin Tip SPE C18 and C8 tips, respectively (ThermoFisher Scientific) before analysis. For phosphosite analysis, 10 μL of digest was injected onto an Acclaim RSLC 120 C18 column (Thermo Fisher Scientific, 2.1 × 100 mm, 2.2 µm, 120 Å) for reversed phase separation at 60°C and 0.4 ml/min, over a linear gradient of 5–40% B over 25 min, 40–90% B over 8 min followed by equilibration at 5% B for 7 min. ESI source conditions were nebuliser pressure of 45 psig, drying gas flow of 5 L/min and gas temperature of 325°C. Sheath gas temperature of 275°C and gas flow of 12 L/min, capillary voltage of 4000V and nozzle voltage of 300V were also applied. Mass spectra were acquired using MassHunter Acquisition software (version B.08.00) over the 100–3000 m/z range, at a rate of 5 spectra/s and 200 ms/spectrum, using standard mass range mode (3200 m/z) with extended dynamic range (2 GHz) and collection of both centroid and profile data. MS/MS fragmentation spectra were acquired over the 100–3000 m/z range, at a rate of 3 spectra/s and 333.3 ms/spectrum. For intact protein analysis,10 μL of desalted protein (~1 mg/ml) was injected onto a Zorbax 300 Å Stable Bond C8 column (Agilent Technologies, 4.6 × 50 mm, 3.5 μM) for reversed phase separation at 60°C and 0.4 mL/min, over a linear gradient of 15–75% B over 14 min, 75–100% B over 11 min followed by post-run equilibration at 15% B for 10 min. ESI source conditions were nebuliser pressure of 45 psig, drying gas flow of 5 L/min and source gas temperature of 325°C were applied. Sheath gas temperature of 400°C and gas flow of 11 L/min, capillary voltage of 3500V and nozzle voltage of 2000V were also used. Mass spectra were acquired using MassHunter Acquisition software (version B.08.00) over a mass range of 100–3000 m/z, at a rate of 1 spectra/s and 1000 ms/spectrum in extended mass range (20000 m/z) at 1 GHz. Acquired MS and MS/MS spectra were analysed using Agilent MassHunter BioConfirm software (version B.10.00) for identification of phosphorylated residues and subsequent intact mass determination with processing of raw data using maximum entropy deconvolution.

### Analytical size exclusion chromatography

Purified ReoM and P-ReoM proteins were run individually on a Superdex 200 Increase 10/300 GL column. 100 µl samples at 1.5 mg/mL were injected onto a column equilibrated in 10 mM HEPES.HCl pH 8.0 and 100 mM NaCl, with a flow of 0.75 mL/min.

## Data Availability

Genome sequences of shg8, shg10, shg12 and LMSW76 were deposited at ENA under study number PRJEB35110 and sample accession numbers ERS3927571 (SAMEA6127277), ERS3927572 (SAMEA6127278), ERS3927573 (SAMEA6127279), and ERS3967687 (SAMEA6167687) respectively. The co-ordinates and structure factors for the crystal structure of ReoM have been deposited at PDBe with accession code 6TIF. The following datasets were generated: WampSRutterZJRismondoJJenningsCEMöllerLLewisRJHalbedelS2020PrkA controls peptidoglycan biosynthesis through the essential phosphorylation of ReoMEuropean Nucleotide ArchivePRJEB3511010.7554/eLife.56048PMC728669032469310 WampSRutterZJRismondoJJenningsCEMöllerLLewisRJHalbedelS2020PrkA controls peptidoglycan biosynthesis through the essential phosphorylation of ReoMProtein Data Bank6TIF10.7554/eLife.56048PMC728669032469310

## Additional files

Supplementary file 1. Key resources table.

Transparent reporting form

## References

[bib1] Arnaud M, Chastanet A, Débarbouillé M (2004). New vector for efficient allelic replacement in naturally Nontransformable, low-GC-content, gram-positive bacteria. Applied and Environmental Microbiology.

[bib2] Banla IL, Kommineni S, Hayward M, Rodrigues M, Palmer KL, Salzman NH, Kristich CJ (2018). Modulators of *Enterococcus faecalis* cell envelope integrity and antimicrobial resistance influence stable colonization of the mammalian gastrointestinal tract. Infection and Immunity.

[bib3] Battye TG, Kontogiannis L, Johnson O, Powell HR, Leslie AG (2011). *iMOSFLM*: a new graphical interface for diffraction-image processing with *MOSFLM*. Acta Crystallographica. Section D, Biological Crystallography.

[bib4] Blake KL, O'Neill AJ, Mengin-Lecreulx D, Henderson PJ, Bostock JM, Dunsmore CJ, Simmons KJ, Fishwick CW, Leeds JA, Chopra I (2009). The nature of *Staphylococcus aureus* MurA and MurZ and approaches for detection of peptidoglycan biosynthesis inhibitors. Molecular Microbiology.

[bib5] Booth S, Lewis RJ (2019). Structural basis for the coordination of cell division with the synthesis of the bacterial cell envelope. Protein Science.

[bib6] Boutte CC, Baer CE, Papavinasasundaram K, Liu W, Chase MR, Meniche X, Fortune SM, Sassetti CM, Ioerger TR, Rubin EJ (2016). A cytoplasmic peptidoglycan amidase homologue controls mycobacterial cell wall synthesis. eLife.

[bib7] Brown ED, Vivas EI, Walsh CT, Kolter R (1995). MurA (MurZ), the enzyme that catalyzes the first committed step in Peptidoglycan Biosynthesis, is essential in *Escherichia coli*. Journal of Bacteriology.

[bib8] Claessen D, Emmins R, Hamoen LW, Daniel RA, Errington J, Edwards DH (2008). Control of the cell elongation-division cycle by shuttling of PBP1 protein in *Bacillus subtilis*. Molecular Microbiology.

[bib9] Cleverley RM, Rismondo J, Lockhart-Cairns MP, Van Bentum PT, Egan AJ, Vollmer W, Halbedel S, Baldock C, Breukink E, Lewis RJ (2016). Subunit arrangement in GpsB, a regulator of cell wall biosynthesis. Microbial Drug Resistance.

[bib10] Cleverley RM, Rutter ZJ, Rismondo J, Corona F, Tsui H-CT, Alatawi FA, Daniel RA, Halbedel S, Massidda O, Winkler ME, Lewis RJ (2019). The cell cycle regulator GpsB functions as cytosolic adaptor for multiple cell wall enzymes. Nature Communications.

[bib11] Cuenot E, Garcia-Garcia T, Douche T, Gorgette O, Courtin P, Denis-Quanquin S, Hoys S, Tremblay YDN, Matondo M, Chapot-Chartier MP, Janoir C, Dupuy B, Candela T, Martin-Verstraete I (2019). The ser/Thr kinase PrkC participates in cell wall homeostasis and antimicrobial resistance in *Clostridium difficile*. Infection and Immunity.

[bib12] Débarbouillé M, Dramsi S, Dussurget O, Nahori MA, Vaganay E, Jouvion G, Cozzone A, Msadek T, Duclos B (2009). Characterization of a serine/threonine kinase involved in virulence of *Staphylococcus aureus*. Journal of Bacteriology.

[bib13] Deng LL, Humphries DE, Arbeit RD, Carlton LE, Smole SC, Carroll JD (2005). Identification of a novel peptidoglycan hydrolase CwlM in *mycobacterium tuberculosis*. Biochimica Et Biophysica Acta (BBA) - Proteins and Proteomics.

[bib14] Dephoure N, Gould KL, Gygi SP, Kellogg DR (2013). Mapping and analysis of phosphorylation sites: a quick guide for cell biologists. Molecular Biology of the Cell.

[bib15] Du W, Brown JR, Sylvester DR, Huang J, Chalker AF, So CY, Holmes DJ, Payne DJ, Wallis NG (2000). Two active forms of UDP-N-acetylglucosamine enolpyruvyl transferase in gram-positive bacteria. Journal of Bacteriology.

[bib16] Dworkin J (2015). Ser/Thr phosphorylation as a regulatory mechanism in bacteria. Current Opinion in Microbiology.

[bib17] Egan AJ, Cleverley RM, Peters K, Lewis RJ, Vollmer W (2017). Regulation of bacterial cell wall growth. The FEBS Journal.

[bib18] Elsholz AK, Turgay K, Michalik S, Hessling B, Gronau K, Oertel D, Mäder U, Bernhardt J, Becher D, Hecker M, Gerth U (2012). Global impact of protein arginine phosphorylation on the physiology of *Bacillus subtilis*. PNAS.

[bib19] Emami K, Guyet A, Kawai Y, Devi J, Wu LJ, Allenby N, Daniel RA, Errington J (2017). RodA as the missing glycosyltransferase in *Bacillus subtilis* and antibiotic discovery for the peptidoglycan polymerase pathway. Nature Microbiology.

[bib20] Emsley P, Lohkamp B, Scott WG, Cowtan K (2010). Features and development of *coot*. Acta Crystallographica. Section D, Biological Crystallography.

[bib21] Evans PR, Murshudov GN (2013). How good are my data and what is the resolution?. Acta Crystallographica Section D Biological Crystallography.

[bib22] Gaidenko TA, Kim TJ, Price CW (2002). The PrpC serine-threonine phosphatase and PrkC kinase have opposing physiological roles in stationary-phase *Bacillus subtilis* cells. Journal of Bacteriology.

[bib23] Gee CL, Papavinasasundaram KG, Blair SR, Baer CE, Falick AM, King DS, Griffin JE, Venghatakrishnan H, Zukauskas A, Wei JR, Dhiman RK, Crick DC, Rubin EJ, Sassetti CM, Alber T (2012). A phosphorylated pseudokinase complex controls cell wall synthesis in mycobacteria. Science Signaling.

[bib24] Gerth U, Kirstein J, Mostertz J, Waldminghaus T, Miethke M, Kock H, Hecker M (2004). Fine-tuning in regulation of clp protein content in *Bacillus subtilis*. Journal of Bacteriology.

[bib25] Gerth U, Kock H, Kusters I, Michalik S, Switzer RL, Hecker M (2008). Clp-dependent proteolysis down-regulates central metabolic pathways in glucose-starved *Bacillus subtilis*. Journal of Bacteriology.

[bib26] Glaser P, Frangeul L, Buchrieser C, Rusniok C, Amend A, Baquero F, Berche P, Bloecker H, Brandt P, Chakraborty T, Charbit A, Chetouani F, Couvé E, de Daruvar A, Dehoux P, Domann E, Domínguez-Bernal G, Duchaud E, Durant L, Dussurget O, Entian KD, Fsihi H, García-del Portillo F, Garrido P, Gautier L, Goebel W, Gómez-López N, Hain T, Hauf J, Jackson D, Jones LM, Kaerst U, Kreft J, Kuhn M, Kunst F, Kurapkat G, Madueno E, Maitournam A, Vicente JM, Ng E, Nedjari H, Nordsiek G, Novella S, de Pablos B, Pérez-Diaz JC, Purcell R, Remmel B, Rose M, Schlueter T, Simoes N, Tierrez A, Vázquez-Boland JA, Voss H, Wehland J, Cossart P (2001). Comparative genomics of *listeria* species. Science.

[bib27] Griffin JE, Gawronski JD, Dejesus MA, Ioerger TR, Akerley BJ, Sassetti CM (2011). High-resolution phenotypic profiling defines genes essential for mycobacterial growth and cholesterol catabolism. PLOS Pathogens.

[bib28] Halbedel S, Lewis RJ (2019). Structural basis for interaction of DivIVA/GpsB proteins with their ligands. Molecular Microbiology.

[bib29] Hall CL, Tschannen M, Worthey EA, Kristich CJ (2013). IreB, a ser/Thr kinase substrate, influences antimicrobial resistance in *Enterococcus faecalis*. Antimicrobial Agents and Chemotherapy.

[bib30] Hall CL, Lytle BL, Jensen D, Hoff JS, Peterson FC, Volkman BF, Kristich CJ (2017). Structure and dimerization of IreB, a negative regulator of cephalosporin resistance in *Enterococcus faecalis*. Journal of Molecular Biology.

[bib31] Hardt P, Engels I, Rausch M, Gajdiss M, Ulm H, Sass P, Ohlsen K, Sahl HG, Bierbaum G, Schneider T, Grein F (2017). The cell wall precursor lipid II acts as a molecular signal for the ser/Thr kinase PknB of *Staphylococcus aureus*. International Journal of Medical Microbiology.

[bib32] Irazoki O, Hernandez SB, Cava F (2019). Peptidoglycan muropeptides: release, perception, and functions as signaling molecules. Frontiers in Microbiology.

[bib33] Johnson LN, Lewis RJ (2001). Structural basis for control by phosphorylation. Chemical Reviews.

[bib34] Karimova G, Pidoux J, Ullmann A, Ladant D (1998). A bacterial two-hybrid system based on a reconstituted signal transduction pathway. PNAS.

[bib35] Kaur P, Rausch M, Malakar B, Watson U, Damle NP, Chawla Y, Srinivasan S, Sharma K, Schneider T, Jhingan GD, Saini D, Mohanty D, Grein F, Nandicoori VK (2019). LipidII interaction with specific residues of *mycobacterium tuberculosis* PknB extracytoplasmic domain governs its optimal activation. Nature Communications.

[bib36] Kennelly PJ (2001). Protein phosphatases--a phylogenetic perspective. Chemical Reviews.

[bib37] Kieser KJ, Boutte CC, Kester JC, Baer CE, Barczak AK, Meniche X, Chao MC, Rego EH, Sassetti CM, Fortune SM, Rubin EJ (2015). Phosphorylation of the peptidoglycan synthase PonA1 governs the rate of polar elongation in mycobacteria. PLOS Pathogens.

[bib38] Kirstein J, Dougan DA, Gerth U, Hecker M, Turgay K (2007). The tyrosine kinase McsB is a regulated adaptor protein for ClpCP. The EMBO Journal.

[bib39] Kirstein J, Molière N, Dougan DA, Turgay K (2009). Adapting the machine: adaptor proteins for Hsp100/Clp and AAA+ proteases. Nature Reviews Microbiology.

[bib40] Kock H, Gerth U, Hecker M (2004). MurAA, catalysing the first committed step in Peptidoglycan Biosynthesis, is a target of Clp-dependent proteolysis in *Bacillus subtilis*. Molecular Microbiology.

[bib41] Kohlrausch U, Höltje JV (1991). Analysis of murein and murein precursors during antibiotic-induced lysis of *Escherichia coli*. Journal of Bacteriology.

[bib42] Koo BM, Kritikos G, Farelli JD, Todor H, Tong K, Kimsey H, Wapinski I, Galardini M, Cabal A, Peters JM, Hachmann AB, Rudner DZ, Allen KN, Typas A, Gross CA (2017). Construction and analysis of two Genome-Scale deletion libraries for *Bacillus subtilis*. Cell Systems.

[bib43] Kristich CJ, Wells CL, Dunny GM (2007). A eukaryotic-type ser/Thr kinase in *Enterococcus faecalis* mediates antimicrobial resistance and intestinal persistence. PNAS.

[bib44] Kristich CJ, Little JL, Hall CL, Hoff JS (2011). Reciprocal regulation of cephalosporin resistance in *Enterococcus faecalis*. mBio.

[bib45] Lewis RJ (2017). The GpsB files: the truth is out there. Molecular Microbiology.

[bib46] Macek B, Mijakovic I, Olsen JV, Gnad F, Kumar C, Jensen PR, Mann M (2007). The Serine/Threonine/Tyrosine Phosphoproteome of the Model Bacterium *Bacillus subtilis*. Molecular & Cellular Proteomics.

[bib47] Madec E, Laszkiewicz A, Iwanicki A, Obuchowski M, Séror S (2002). Characterization of a membrane-linked ser/Thr protein kinase in *Bacillus subtilis*, implicated in developmental processes. Molecular Microbiology.

[bib48] Madec E, Stensballe A, Kjellström S, Cladière L, Obuchowski M, Jensen ON, Séror SJ (2003). Mass spectrometry and site-directed mutagenesis identify several autophosphorylated residues required for the activity of PrkC, a ser/Thr kinase from *Bacillus subtilis*. Journal of Molecular Biology.

[bib49] Manuse S, Fleurie A, Zucchini L, Lesterlin C, Grangeasse C (2016). Role of eukaryotic-like serine/threonine kinases in bacterial cell division and morphogenesis. FEMS Microbiology Reviews.

[bib50] Marston AL, Thomaides HB, Edwards DH, Sharpe ME, Errington J (1998). Polar localization of the MinD protein of *Bacillus subtilis* and its role in selection of the mid-cell division site. Genes & Development.

[bib51] McCoy AJ, Grosse-Kunstleve RW, Adams PD, Winn MD, Storoni LC, Read RJ (2007). Phaser crystallographic software. Journal of Applied Crystallography.

[bib52] Meeske AJ, Sham LT, Kimsey H, Koo BM, Gross CA, Bernhardt TG, Rudner DZ (2015). MurJ and a novel lipid II flippase are required for cell wall biogenesis in *Bacillus subtilis*. PNAS.

[bib53] Meeske AJ, Riley EP, Robins WP, Uehara T, Mekalanos JJ, Kahne D, Walker S, Kruse AC, Bernhardt TG, Rudner DZ (2016). SEDS proteins are a widespread family of bacterial cell wall polymerases. Nature.

[bib54] Mir M, Asong J, Li X, Cardot J, Boons GJ, Husson RN (2011). The extracytoplasmic domain of the *mycobacterium tuberculosis* ser/Thr kinase PknB binds specific muropeptides and is required for PknB localization. PLOS Pathogens.

[bib55] Misra SK, Milohanic E, Aké F, Mijakovic I, Deutscher J, Monnet V, Henry C (2011). Analysis of the serine/threonine/tyrosine phosphoproteome of the pathogenic bacterium *listeria monocytogenes* reveals phosphorylated proteins related to virulence. Proteomics.

[bib56] Molière N, Turgay K (2009). Chaperone-protease systems in regulation and protein quality control in *Bacillus subtilis*. Research in Microbiology.

[bib57] Monk IR, Gahan CG, Hill C (2008). Tools for functional postgenomic analysis of *listeria monocytogenes*. Applied and Environmental Microbiology.

[bib58] Moynihan PJ, Sychantha D, Clarke AJ (2014). Chemical biology of peptidoglycan acetylation and deacetylation. Bioorganic Chemistry.

[bib59] Mulvenna N, Hantke I, Burchell L, Nicod S, Bell D, Turgay K, Wigneshweraraj S (2019). Xenogeneic modulation of the ClpCP protease of *Bacillus subtilis* by a phage-encoded adaptor-like protein. Journal of Biological Chemistry.

[bib60] Murshudov GN, Vagin AA, Dodson EJ (1997). Refinement of macromolecular structures by the maximum-likelihood method. Acta Crystallographica Section D Biological Crystallography.

[bib61] Nováková L, Sasková L, Pallová P, Janecek J, Novotná J, Ulrych A, Echenique J, Trombe MC, Branny P (2005). Characterization of a eukaryotic type serine/threonine protein kinase and protein phosphatase of *streptococcus pneumoniae* and identification of kinase substrates. FEBS Journal.

[bib62] Parikh A, Verma SK, Khan S, Prakash B, Nandicoori VK (2009). PknB-mediated phosphorylation of a novel substrate, N-acetylglucosamine-1-phosphate uridyltransferase, modulates its acetyltransferase activity. Journal of Molecular Biology.

[bib63] Pensinger DA, Aliota MT, Schaenzer AJ, Boldon KM, Ansari IH, Vincent WJB, Knight B, Reniere ML, Striker R, Sauer J-D (2014). Selective pharmacologic inhibition of a PASTA kinase increases *Listeria monocytogenes* Susceptibility to β-Lactam Antibiotics. Antimicrobial Agents and Chemotherapy.

[bib64] Pensinger DA, Boldon KM, Chen GY, Vincent WJB, Sherman K, Xiong M, Schaenzer AJ, Forster ER, Coers J, Striker R, Sauer J-D (2016). The *listeria monocytogenes* PASTA Kinase PrkA and Its Substrate YvcK Are Required for Cell Wall Homeostasis, Metabolism, and Virulence. PLOS Pathogens.

[bib65] Peränen J, Rikkonen M, Hyvönen M, Kääriäinen L (1996). T7 vectors with modified T7lac promoter for expression of proteins *in Escherichia coli*. Analytical Biochemistry.

[bib66] Pompeo F, Foulquier E, Serrano B, Grangeasse C, Galinier A (2015). Phosphorylation of the cell division protein GpsB regulates PrkC kinase activity through a negative feedback loop in *Bacillus subtilis*. Molecular Microbiology.

[bib67] Rakette S, Donat S, Ohlsen K, Stehle T (2012). Structural analysis of *Staphylococcus aureus* serine/threonine kinase PknB. PLOS ONE.

[bib68] Ravikumar V, Shi L, Krug K, Derouiche A, Jers C, Cousin C, Kobir A, Mijakovic I, Macek B (2014). Quantitative phosphoproteome analysis of *Bacillus subtilis* reveals novel substrates of the kinase PrkC and phosphatase PrpC. Molecular & Cellular Proteomics.

[bib69] Rismondo J, Möller L, Aldridge C, Gray J, Vollmer W, Halbedel S (2015). Discrete and overlapping functions of peptidoglycan synthases in growth, cell division and virulence of *listeria monocytogenes*. Molecular Microbiology.

[bib70] Rismondo J, Cleverley RM, Lane HV, Großhennig S, Steglich A, Möller L, Mannala GK, Hain T, Lewis RJ, Halbedel S (2016). Structure of the bacterial cell division determinant GpsB and its interaction with penicillin-binding proteins. Molecular Microbiology.

[bib71] Rismondo J, Bender JK, Halbedel S (2017). Suppressor mutations linking *gpsB* with the first committed step of peptidoglycan biosynthesis in *listeria monocytogenes*. Journal of Bacteriology.

[bib72] Rouquette C, Ripio MT, Pellegrini E, Bolla JM, Tascon RI, Vázquez-Boland JA, Berche P (1996). Identification of a ClpC ATPase required for stress tolerance and *in vivo* survival of *listeria monocytogenes*. Molecular Microbiology.

[bib73] Rued BE, Zheng JJ, Mura A, Tsui HT, Boersma MJ, Mazny JL, Corona F, Perez AJ, Fadda D, Doubravová L, Buriánková K, Branny P, Massidda O, Winkler ME (2017). Suppression and synthetic-lethal genetic relationships of δgpsb mutations indicate that GpsB mediates protein phosphorylation and penicillin-binding protein interactions in *streptococcus pneumoniae* D39. Molecular Microbiology.

[bib74] Ruiz N (2008). Bioinformatics identification of MurJ (MviN) as the peptidoglycan lipid II flippase in *Escherichia coli*. PNAS.

[bib75] Sambrook J, Fritsch EF, Maniatis T (1989). Molecular Cloning: A Laboratory Manual.

[bib76] Sapkota M, Marreddy RKR, Wu X, Kumar M, Hurdle JG (2020). The early stage peptidoglycan biosynthesis mur enzymes are antibacterial and antisporulation drug targets for recurrent *Clostridioides difficile* infection. Anaerobe.

[bib77] Sauvage E, Kerff F, Terrak M, Ayala JA, Charlier P (2008). The penicillin-binding proteins: structure and role in peptidoglycan biosynthesis. FEMS Microbiology Reviews.

[bib78] Shah IM, Laaberki MH, Popham DL, Dworkin J (2008). A eukaryotic-like ser/Thr kinase signals bacteria to exit dormancy in response to peptidoglycan fragments. Cell.

[bib79] Sham LT, Butler EK, Lebar MD, Kahne D, Bernhardt TG, Ruiz N (2014). Bacterial cell wall MurJ is the flippase of lipid-linked precursors for peptidoglycan biogenesis. Science.

[bib80] Taguchi A, Welsh MA, Marmont LS, Lee W, Sjodt M, Kruse AC, Kahne D, Bernhardt TG, Walker S (2019). FtsW is a peptidoglycan polymerase that is functional only in complex with its cognate penicillin-binding protein. Nature Microbiology.

[bib81] Trentini DB, Suskiewicz MJ, Heuck A, Kurzbauer R, Deszcz L, Mechtler K, Clausen T (2016). Arginine phosphorylation marks proteins for degradation by a clp protease. Nature.

[bib82] Turapov O, Forti F, Kadhim B, Ghisotti D, Sassine J, Straatman-Iwanowska A, Bottrill AR, Moynihan PJ, Wallis R, Barthe P, Cohen-Gonsaud M, Ajuh P, Vollmer W, Mukamolova GV (2018). Two faces of CwlM, an essential PknB substrate, in *mycobacterium tuberculosis*. Cell Reports.

[bib83] Typas A, Banzhaf M, Gross CA, Vollmer W (2012). From the regulation of peptidoglycan synthesis to bacterial growth and morphology. Nature Reviews Microbiology.

[bib84] Uehara T, Bernhardt TG (2011). More than just lysins: peptidoglycan hydrolases tailor the cell wall. Current Opinion in Microbiology.

[bib85] Ulrych A, Holečková N, Goldová J, Doubravová L, Benada O, Kofroňová O, Halada P, Branny P (2016). Characterization of pneumococcal ser/Thr protein phosphatase *phpP* mutant and identification of a novel *PhpP* substrate, putative RNA binding protein jag. BMC Microbiology.

[bib86] van den Ent F, Löwe J (2006). RF cloning: a restriction-free method for inserting target genes into plasmids. Journal of Biochemical and Biophysical Methods.

[bib87] Vesić D, Kristich CJ (2012). MurAA is required for intrinsic cephalosporin resistance of *Enterococcus faecalis*. Antimicrobial Agents and Chemotherapy.

[bib88] Vollmer W, Blanot D, de Pedro MA (2008a). Peptidoglycan structure and architecture. FEMS Microbiology Reviews.

[bib89] Vollmer W, Joris B, Charlier P, Foster S (2008b). Bacterial peptidoglycan (murein) hydrolases. FEMS Microbiology Reviews.

