# Supplementary material for: PrkA controls peptidoglycan biosynthesis through the essential phosphorylation of ReoM
Source: eLife. 2020 May 29;9:e56048. doi: 10.7554/eLife.56048 (PMC7286690; doi:10.7554/eLife.56048)
Supplement: Supplementary file 1. [file elife-56048-supp1.docx]

| **Key Resources Table** | | | | |
| --- | --- | --- | --- | --- |
| **Reagent type (species) or resource** | **Designation** | **Source or reference** | **Identifiers** | **Additional information** |
| antibody | anti-ClpC*^Bsu^* antibody (Rabbit polyclonal) | PMID:14679237 |  | dilution (1:10000), crossreacts with ClpC*^Lmo^* |
| antibody | anti-DivIVA*^Bsu^* antibody (Rabbit polyclonal) | PMID:9808628 |  | dilution (1:5000), crossreacts with DivIVA*^Lmo^* |
| antibody | anti-GlmS*^Bsu^* antibody (Rabbit polyclonal) | PMID:17981983 |  | dilution (1:5000) |
| antibody | anti-IlvB*^Bsu^* antibody (Rabbit polyclonal) | PMID:17981983 |  | dilution (1:5000) |
| antibody | anti-MurAA*^Bsu^* antibody (Rabbit polyclonal) | PMID:14763982 |  | dilution (1:5000), crossreacts with MurA*^Lmo^* |
| antibody | anti-rabbit IgG (whole molecule)-peroxidase antibody produced in goat | Sigma-Aldrich | RRID:AB_257896 |  |
| chemical compound or drug | ampicillin sodium salt | Sigma-Aldrich | A9518-5G |  |
| chemical compound or drug | ATP | Sigma-Aldrich | A7699-10G |  |
| chemical compound or drug | DNase I (Roche) | Sigma Aldrich | 10104159001 |  |
| chemical compound or drug | erythromycin | Sigma-Aldrich | E5389-5G |  |
| chemical compound or drug | IPTG | ForMedium | IPTG025 |  |
| chemical compound or drug | Kanamycin | ForMedium | Kan0025 |  |
| chemical compound or drug | X-Gal | peqLab | 37-2610 |  |
| chemical compound, drug | ceftriaxone E-test strips 0.016 - 256 µg/ml | BestbionDX | 92042 |  |
| chemical compound, drug | nile red | Sigma Aldrich | N3013-100MG |  |
| commercial assay or kit | 5 mL Ni-NTA superflow cartridge | Qiagen | Cat No./ID: 30761 |  |
| commercial assay or kit | 96 well MRC crystallisation plates | Molecular Dimensions | MD11-00-10 |  |
| commercial assay or kit | Acclaim RSLC 120 C18 column | ThermoFisher | P/N: 68982 |  |
| commercial assay or kit | Bacterial Adenylate Cyclase Two-Hybrid System Kit | Euromedex | EUK001 |  |
| commercial assay or kit | HyperSep SpinTip SPE C18 extraction tips | Thermo Fisher | 60109-412 |  |
| commercial assay or kit | HyperSep SpinTip SPE C8 extraction tips | Thermo Fisher | 60109-413 |  |
| commercial assay or kit | JCSG+ Crystallisation screen | Molecular Dimensions |  |  |
| commercial assay or kit | MiSeq Reagent Kit v3 cartridge (600-cycle kit) | Illumina | MS-102-3003 |  |
| commercial assay or kit | MonoQ 5/50 GL column | GE Healthcare | 17516601 |  |
| commercial assay or kit | Mosquito | TTP Labtech |  |  |
| commercial assay or kit | Nextera XT DNA Library Prep Kit | Illumina | FC-131-1024 |  |
| commercial assay or kit | PD 10 desalting column | GE Healthcare | 17085101 |  |
| commercial assay or kit | Rayon loops | Hampton Research |  |  |
| commercial assay or kit | Smart Digest Soluble Trypsin Kit | Thermo Fisher | 60113-101 |  |
| commercial assay or kit | Superdex 200 increase 10/300 GL column | GE Healthcare | 28990944 |  |
| commercial assay or kit | Superdex 200 XK16/60 | GE Healthcare |  |  |
| commercial assay or kit | Superdex 75 XK16/60 | GE Healthcare |  |  |
| commercial assay or kit | SuperSignal™ West Dura chemiluminescence substrate | Thermo Scientific | 34076 |  |
| commercial assay or kit | Zorbax 300Å Stable Bond C8 column | Agilent | P/N: 865973-906 |  |
| gene (*L. monocytogenes*) | *gpsB* (*lmo1888*) | *L. monocytogenes* EGD-e | NCBI Gene ID: 985801 |  |
| gene (*L. monocytogenes*) | *reoM* (*lmo1503*) | *L. monocytogenes* EGD-e | NCBI Gene ID: 987746 |  |
| gene (*L. monocytogenes*) | *reoY* (*lmo1921*) | *L. monocytogenes* EGD-e | NCBI Gene ID: 985800 |  |
| gene (*L. monocytogenes*) | *murZ* (*lmo2552*) | *L. monocytogenes* EGD-e | NCBI Gene ID: 987263 |  |
| gene (*L. monocytogenes*) | *murA*  (*lmo2526*) | *L. monocytogenes* EGD-e | NCBI Gene ID: 984514 |  |
| gene (*L. monocytogenes*) | *clpC*  (*lmo0232*) | *L. monocytogenes* EGD-e | NCBI Gene ID: 987203 |  |
| gene (*L. monocytogenes*) | *prkA*  (*lmo1820*) | *L. monocytogenes* EGD-e | NCBI Gene ID: 985900 |  |
| gene (*L. monocytogenes*) | *prpC*  (*lmo1821*) | *L. monocytogenes* EGD-e | NCBI Gene ID: 985901 |  |
| peptide, recombinant protein | BamHI | NEB | R3136S |  |
| peptide, recombinant protein | BglII | NEB | R0144S |  |
| peptide, recombinant protein | EcoRI | NEB | R3101S |  |
| peptide, recombinant protein | KpnI | NEB | R3142S |  |
| peptide, recombinant protein | PstI | NEB | R3140S |  |
| peptide, recombinant protein | NcoI | Thermo Fisher Scientific | Cat# ER0571 |  |
| peptide, recombinant protein | SalI | NEB | R3138S |  |
| peptide, recombinant protein | XbaI | NEB | R0145S |  |
| peptide, recombinant protein | XhoI | Thermo Fisher Scientific | Cat# ER0695 |  |
| recombinant DNA reagent | p25-N (plasmid) | PMID:9576956 |  |  |
| recombinant DNA reagent | pETM11 (plasmid) | PMID:8660525 |  |  |
| recombinant DNA reagent | pIMK3 (plasmid) | PMID:18441118 |  |  |
| recombinant DNA reagent | pKT25 (plasmid) | PMID:9576956 |  |  |
| recombinant DNA reagent | pMAD (plasmid) | PMID:15528558 |  |  |
| recombinant DNA reagent | pRK793 (plasmid) |  | RRID:Addgene_8827 | expresses His-tagged TEV protease |
| recombinant DNA reagent | pUT18 (plasmid) | PMID:9576956 |  |  |
| recombinant DNA reagent | pUT18C (plasmid) | PMID:9576956 |  |  |
| sequence-based-reagent | JR163 | Integrated DNA Technologies | PCR primers | GCGCCCATGGCTAAGGCATCCATTTCAATAGACGAGAAG |
| sequence-based-reagent | JR164 | Integrated DNA Technologies | PCR primers | GCGCGTCGACTTATTCTTTTTCCGTATCCATTTGCTGTA |
| sequence-based-reagent | JR169 | Integrated DNA Technologies | PCR primers | GCGCCCATGGATTCAAAAGATCAAACAATGTTTTACAACTTC |
| sequence-based-reagent | JR170 | Integrated DNA Technologies | PCR primers | GCGCGTCGACTCATTTCTCACCAATTTCGTTATTTTTCAG |
| sequence-based-reagent | JR197 | Integrated DNA Technologies | PCR primers | GCGCGGATCCCAATTATTTCGAATGGTGCGGTGTC |
| sequence-based-reagent | JR198 | Integrated DNA Technologies | PCR primers | TCCTTATTCGTCGACCATCTTTCCTCAGTCCCTTCCTG |
| sequence-based-reagent | JR199 | Integrated DNA Technologies | PCR primers | GGAAAGATGGTCGACGAATAAGGAATAAATCCTAGTTAGTAGGG |
| sequence-based-reagent | JR200 | Integrated DNA Technologies | PCR primers | CGCGCGAATTCCCAAGACTCAACCTCTTTCACTC |
| sequence-based-reagent | JR249 | Integrated DNA Technologies | PCR primers | GCGCCTGCAGAAAAAATTATTGTACGCGGTGGAAAAC |
| sequence-based-reagent | JR250 | Integrated DNA Technologies | PCR primers | GCGCGGTACCGCGAATAAAGACGCTAAGTTTGTTACATCG |
| sequence-based-reagent | JR253 | Integrated DNA Technologies | PCR primers | GCGCTCTAGAAAAGGCATCCATTTCAATAGACGAG |
| sequence-based-reagent | JR254 | Integrated DNA Technologies | PCR primers | GCGCGGTACCTCTTTTTCCGTATCCATTTGCTG |
| sequence-based-reagent | JR255 | Integrated DNA Technologies | PCR primers | GCGCTCTAGATTCAAAAGATCAAACAATGTTTTACAAC |
| sequence-based-reagent | JR256 | Integrated DNA Technologies | PCR primers | GCGCGGTACCTTCTCACCAATTTCGTTATTTTTCAG |
| sequence-based-reagent | JR257 | Integrated DNA Technologies | PCR primers | GCGCCTGCAGGGAAAAAATTATTGTACGCGGTGGAAAAC |
| sequence-based-reagent | JR264 | Integrated DNA Technologies | PCR primers | GCGCAGATCTGGCAAATACAGCATTGAACTATGTG |
| sequence-based-reagent | JR265 | Integrated DNA Technologies | PCR primers | GCGCGGATCCAATCGAAGCACCTCATTCCTTC |
| sequence-based-reagent | JR266 | Integrated DNA Technologies | PCR primers | GCGCGGATCCATGAGAATAATGGGTTTAGATGTCGGC |
| sequence-based-reagent | JR267 | Integrated DNA Technologies | PCR primers | GCGCGTCGACGCTAGGAATGTAGCAAGGATTTCTTC |
| sequence-based-reagent | SHW815 | Integrated DNA Technologies | PCR primers | GATCTATCGATGCATGCCATGGGCTAAATGACCAAGGAATTACCG |
| sequence-based-reagent | SHW816 | Integrated DNA Technologies | PCR primers | CGCGTCGGGCGATATCGGATCCTTTCTTCCGCGTTTTGGTAACG |
| sequence-based-reagent | SHW817 | Integrated DNA Technologies | PCR primers | CAATCATCATTTTAAAAGCACCTCACTATTTTTCAG |
| sequence-based-reagent | SHW818 | Integrated DNA Technologies | PCR primers | TGCTTTTAAAATGATGATTGGTAAGCGATTAAGC |
| sequence-based-reagent | SHW819 | Integrated DNA Technologies | PCR primers | GATCTATCGATGCATGCCATGGAGATAGAGGCAGAATAAGACATC |
| sequence-based-reagent | SHW820 | Integrated DNA Technologies | PCR primers | CGCGTCGGGCGATATCGGATCCGGTATTTACAACCACTACGTCG |
| sequence-based-reagent | SHW821 | Integrated DNA Technologies | PCR primers | CGTTCTtaTTTCATGAAGCATCCCTCCCTTTC |
| sequence-based-reagent | SHW822 | Integrated DNA Technologies | PCR primers | TGCTTCATGAAAtaAGAACGGAGGAAATGTGCTG |
| sequence-based-reagent | SHW830 | Integrated DNA Technologies | PCR primers | gcgcgcTCTAGAtggacgatttacgcaaagagctcag |
| sequence-based-reagent | SHW831 | Integrated DNA Technologies | PCR primers | gcgcgcGGTACCttagcttttacttttttagaggttgttttc |
| sequence-based-reagent | SHW832 | Integrated DNA Technologies | PCR primers | gcgcgcTCTAGAaattccaacagtaattgaacaaactagc |
| sequence-based-reagent | SHW833 | Integrated DNA Technologies | PCR primers | gcgcgcGGTACCccttttaagccagatttattaatgataatatc |
| sequence-based-reagent | SW77 | Integrated DNA Technologies | PCR primers | GTAAAACATTGcTTGATCTTTTGAATCCATGGGTTTCAC |
| sequence-based-reagent | SW78 | Integrated DNA Technologies | PCR primers | GATCAAgCAATGTTTTACAACTTCGGCGATGATTC |
| sequence-based-reagent | SW79 | Integrated DNA Technologies | PCR primers | GTAAAACATgtcTTGATCTTTTGAATCCATGGGTTTCAC |
| sequence-based-reagent | SW80 | Integrated DNA Technologies | PCR primers | GATCAAgacATGTTTTACAACTTCGGCG ATGATTC |
| sequence-based-reagent | SW110 | Integrated DNA Technologies | PCR primers | GCGCGCggatccATGCATGCAGAATTTAGAACAGATAG |
| sequence-based-reagent | SW111 | Integrated DNA Technologies | PCR primers | GCGCGCgtcgacTCATGAAGCATCCCTCCCTTTC |
| sequence-based-reagent | SW112 | Integrated DNA Technologies | PCR primers | GCGCGCggatccATGATGATTGGTAAGCGATTAAGCG |
| sequence-based-reagent | SW113 | Integrated DNA Technologies | PCR primers | GCGCGCgtcgacTTAATTTGGATAAGGGACTGTACCTTC |
| sequence-based-reagent | SW136 | Integrated DNA Technologies | PCR primers | CTAAACGagcTATCATACTTCTAGCATCCTTGTGAC |
| sequence-based-reagent | SW137 | Integrated DNA Technologies | PCR primers | GTATGATAgctCGTTTAGAACGAGATGAAATTATCGAG |
| sequence-based-reagent | SW138 | Integrated DNA Technologies | PCR primers | AATTTCATCtgcTTCTAAACGACGTATCATACTTCTAGC |
| sequence-based-reagent | SW139 | Integrated DNA Technologies | PCR primers | GTTTAGAAgcaGATGAAATTATCGAGGAACTTGTCAAAG |
| sequence-based-reagent | SW144 | Integrated DNA Technologies | PCR primers | CCTTGTGagcAGGAATATAAGCAGGATCGCCTG |
| sequence-based-reagent | SW145 | Integrated DNA Technologies | PCR primers | TATATTCCTgctCACAAGGATGCTAGAAGTATGATAC |
| sequence-based-reagent | SW146 | Integrated DNA Technologies | PCR primers | GTATCATACTtgcAGCATCCTTGTGACGAGGAATATAAG |
| sequence-based-reagent | SW147 | Integrated DNA Technologies | PCR primers | GGATGCTgcaAGTATGATACGTCGTTTAGAACGAG |
| sequence-based-reagent | Lmo1503F | Eurofins Genomics | PCR primers | GCTATACCATGGATTCAAAAGATCAAACAATGTTTTACAAC |
| sequence-based-reagent | Lmo1503R | Eurofins Genomics | PCR primers | CGATATCTCGAGTCATTTCTCACCAATTTCGTTATTTTTCAG |
| sequence-based-reagent | PrkAF | Eurofins Genomics | PCR primers | GCTATACCATGGCAATGATGATTGGTAAGCGATTAAGCG |
| sequence-based-reagent | PrkAR | Eurofins Genomics | PCR primers | CGATATCTCGAGTCATTTTTTCTTTTTCTTATCTTTTTTCTCCTCAGG |
| sequence-based-reagent | PrpCF | Eurofins Genomics | PCR primers | GCTATACCATGGCAATGCATGCAGAATTTAGAACAGATAGAG |
| sequence-based-reagent | PrpCR | Eurofins Genomics | PCR primers | CGATATCTCGAGTCATGAAGCATCCCTCCCTTTC |
| software, algorithm | AIMLESS | PMID:23793146 | RRID:SCR_015747 |  |
| software, algorithm | COOT | PMID:20383002 | RRID:SCR_014222 |  |
| software, algorithm | Geneious prime 2020.0.5 | Biomatters Ltd. | RRID:SCR_010519 |  |
| software, algorithm | MassHunter Acquisition | Agilent | Version B.08.0 |  |
| software, algorithm | MassHunter BioConfirm | Agilent | Version B.10.0 |  |
| software, algorithm | MOSFLM | PMID:21460445 |  |  |
| software, algorithm | PHASER | PMID:19461840 |  |  |
| software, algorithm | REFMAC | PMID:15299926 | RRID:SCR_014225 |  |
| strain, strain background  (*L. monocytogenes)* | EGD-e | PMID:11679669 |  |  |
| strain, strain background  (*L. monocytogenes)* | LMJR19 | PMID:26575090 |  | Δ*gpsB (lmo1888)* |
| strain, strain background  (*L. monocytogenes)* | LMJR104 | PMID:27795316 |  | ∆*murZ (lmo2552)* |
| strain, strain background  (*L. monocytogenes)* | LMJR116 | PMID:27795316 |  | *attB::*P*_help_-lacO-murA lacI neo* |
| strain, strain background  (*L. monocytogenes)* | LMJR123 | PMID:27795316 |  | Δ*murA* *(lmo2526) attB::*P*_help_-lacO-murA lacI neo* |
| strain, strain background  (*L. monocytogenes)* | LMJR138 | PMID:27795316 |  | Δ*clpC (lmo0232)* |
| strain, strain background  (*L. monocytogenes)* | *shg8* | this paper | ID_ ENA: SAMEA6127277 | Δ*gpsB reoY* H87Y; see Table 2 |
| strain, strain background  (*L. monocytogenes)* | *shg10* | this paper | ID_ ENA: SAMEA6127278 | Δ*gpsB reoY* TAA74; see Table 2 |
| strain, strain background  (*L. monocytogenes)* | *shg12* | this paper | ID_ ENA: SAMEA6127279 | Δ*gpsB reoM* RBS mutation; see Table 2 |
| strain, strain background  (*L. monocytogenes)* | LMJR96 | this paper |  | ∆*gpsB attB::*P*_help_-lacO-reoM lacI neo*; see Table 2 |
| strain  (*L. monocytogenes)* | LMJR102 | this paper |  | *attB::*P*_help_-lacO-reoM lacI neo*; see Table 2 |
| strain  (*L. monocytogenes)* | LMJR106 | this paper |  | ∆*gpsB attB::*P*_help_-lacO-reoY lacI neo*; see Table 2 |
| strain, strain background  (*L. monocytogenes)* | LMJR120 | this paper |  | Δ*gpsB* Δ*reoY*; see Table 2 |
| strain, strain background  (*L. monocytogenes)* | LMJR137 | this paper |  | Δ*gpsB* Δ*reoM*; see Table 2 |
| strain, strain background  (*L. monocytogenes)* | LMJR171 | this paper |  | Δ*clpC* Δ*murZ*; see Table 2 |
| strain  (*L. monocytogenes)* | LMSW30 | this paper |  | Δ*reoM (lmo1503)*; see Table 2 |
| strain, strain background  (*L. monocytogenes)* | LMSW32 | this paper |  | Δ*reoY (lmo1921)*; see Table 2 |
| strain, strain background  (*L. monocytogenes)* | LMSW50 | this paper |  | Δ*clpC* Δ*reoM*; see Table 2 |
| strain, strain background  (*L. monocytogenes)* | LMSW51 | this paper |  | Δ*clpC* Δ*reoY*; see Table 2 |
| strain, strain background  (*L. monocytogenes)* | LMSW52 | this paper |  | Δ*reoM* *attB::*P*_help_-lacO-reoM T7A lacI neo*; see Table 2 |
| strain, strain background  (*L. monocytogenes)* | LMSW53 | this paper |  | Δ*reoM* *attB::*P*_help_-lacO-reoM T7D lacI neo*; see Table 2 |
| strain, strain background  (*L. monocytogenes)* | LMSW57 | this paper |  | Δ*reoM* *attB::*P*_help_-lacO-reoM lacI neo*; see Table 2 |
| strain, strain background  (*L. monocytogenes)* | LMSW72 | this paper |  | Δ*reoM* *attB::*P*_help_-lacO-reoM T7A lacI neo* Δ*clpC*; see Table 2 |
| strain, strain background  (*L. monocytogenes)* | LMSW76 | this paper | ID_ENA:  SAMEA6167687 | Δ*prpC prkA**; see Table 2 |
| strain, strain background  (*L. monocytogenes)* | LMSW80 | this paper |  | *attB::*P*_help_-lacO-prkA lacI neo*; see Table 2 |
| strain, strain background  (*L. monocytogenes)* | LMSW81 | this paper |  | *attB::*P*_help_-lacO-prpC lacI neo*; see Table 2 |
| strain, strain background  (*L. monocytogenes)* | LMSW83 | this paper |  | Δ*prpC* *attB::*P*_help_-lacO-prpC lacI neo*; see Table 2 |
| strain, strain background  (*L. monocytogenes)* | LMSW84 | this paper |  | Δ*prkA attB::*P*_help_-lacO-prkA lacI neo*; see Table 2 |
| strain, strain background  (*L. monocytogenes)* | LMSW89 | this paper |  | Δ*prkA attB::*P*_help_-lacO-prkA lacI neo* Δ*reoM*; see Table 2 |
| strain, strain background  (*L. monocytogenes)* | LMSW90 | this paper |  | Δ*prkA attB::*P*_help_-lacO-prkA lacI neo* Δ*reoY*; see Table 2 |
| strain, strain background  (*L. monocytogenes)* | LMSW91 | this paper |  | Δ*prkA attB::*P*_help_-lacO-prkA lacI neo* Δ*clpC*; see Table 2 |
| strain, strain background  (*L. monocytogenes)* | LMSW117 | this paper |  | Δ*reoM* Δ*reoY*; see Table 2 |
| strain, strain background  (*L. monocytogenes)* | LMSW118 | this paper |  | Δ*reoY* Δ*murZ*; see Table 2 |
| strain, strain background  (*L. monocytogenes)* | LMSW119 | this paper |  | Δ*reoM* Δ*murZ*; see Table 2 |
| strain, strain background  (*L. monocytogenes)* | LMSW120 | this paper |  | Δ*reoM* *attB::*P*_help_-lacO-reoM R66A lacI neo*; see Table 2 |
| strain  (*L. monocytogenes)* | LMSW121 | this paper |  | Δ*reoM* *attB::*P*_help_-lacO-reoM R70A lacI neo*; see Table 2 |
| strain, strain background  (*L. monocytogenes)* | LMSW123 | this paper |  | Δ*reoM* *attB::*P*_help_-lacO-reoM T7A lacI neo* Δ*reoY*; see Table 2 |
| strain, strain background  (*L. monocytogenes)* | LMSW124 | this paper |  | Δ*reoM* *attB::*P*_help_-lacO-reoM T7A lacI neo* Δ*murZ*; see Table 2 |
| strain, strain background  (*L. monocytogenes)* | LMSW125 | this paper |  | Δ*reoM* *attB::*P*_help_-lacO-reoM R57A lacI neo*; see Table 2 |
| strain, strain background  (*L. monocytogenes)* | LMSW126 | this paper |  | Δ*reoM* *attB::*P*_help_-lacO-reoM R62A lacI neo*; see Table 2 |
| strain, strain background  (*L. monocytogenes)* | LMSW138 | this paper |  | Δ*reoY*  *attB::*P*_help_-lacO-reoY lacI neo*; see Table 2 |
| strain, strain background  (*L. monocytogenes)* | LMSW139 | this paper |  | Δ*murZ*  *attB::*P*_help_-lacO-murZ lacI neo*; see Table 2 |
| strain, strain background  (*Escherichia coli*) | TOP10 | ThermoFischer Scientific | C404003 |  |
| strain, strain background  (*E. coli*) | BL21 (DE3) | NEB |  | competent cells |
| strain, strain background  (*Bacillus subtilis*) | 168 | other |  | lab stock |
| strain, strain background  (*B. subtilis*) | BKE00860 | PMID:28189581;National BioResource Project (NIG, Japan) |  | Δ*clpC* |
| strain, strain background  (*B. subtilis*) | BKE22180 | PMID:28189581;National BioResource Project (NIG, Japan) |  | Δ*gpsB* |
| strain, strain background  (*B. subtilis*) | BKE22580 | PMID:28189581;National BioResource Project (NIG, Japan) |  | Δ*ypiB (reoY)* |
| strain, strain background  (*B. subtilis*) | BKE27400 | PMID:28189581;National BioResource Project (NIG, Japan) |  | Δ*yrzL (reoM)* |
