# Supplementary material for: PrkA controls peptidoglycan biosynthesis through the essential phosphorylation of ReoM
Source: eLife. 2020 May 29;9:e56048. doi: 10.7554/eLife.56048 (PMC7286690; doi:10.7554/eLife.56048)
Supplement: Transparent reporting form [file elife-56048-transrepform.docx]

***eLife’s* transparent reporting form**

We encourage authors to provide detailed information *within their submission* to facilitate the interpretation and replication of experiments. Authors can upload supporting documentation to indicate the use of appropriate reporting guidelines for health-related research (see [EQUATOR Network](http://www.equator-network.org/%20)), life science research (see the [BioSharing Information Resource](https://biosharing.org/)), or the [ARRIVE guidelines](http://www.plosbiology.org/article/info:doi/10.1371/journal.pbio.1000412) for reporting work involving animal research. Where applicable, authors should refer to any relevant reporting standards documents in this form.

If you have any questions, please consult our Journal Policies and/or contact us: [editorial@elifesciences.org](mailto:editorial@elifesciences.org).

**Sample-size estimation**

- You should state whether an appropriate sample size was computed when the study was being designed
- You should state the statistical method of sample size computation and any required assumptions
- If no explicit power analysis was used, you should describe how you decided what sample (replicate) size (number) to use

Please outline where this information can be found within the submission (e.g., sections or figure legends), or explain why this information doesn’t apply to your submission:

No explicit power analysis was used. We used a standard sample size of three replicates in all experiments, if not stated otherwise. For cell length measurements of bacterial cells, average cell lengths were calculated from 300 bacterial cells. Information on sample sizes is given in the figure legends.

**Replicates**

- You should report how often each experiment was performed
- You should include a definition of biological versus technical replication
- The data obtained should be provided and sufficient information should be provided to indicate the number of independent biological and/or technical replicates
- If you encountered any outliers, you should describe how these were handled
- Criteria for exclusion/inclusion of data should be clearly stated
- High-throughput sequence data should be uploaded before submission, with a private link for reviewers provided (these are available from both GEO and ArrayExpress)

Please outline where this information can be found within the submission (e.g., sections or figure legends), or explain why this information doesn’t apply to your submission:

All experiments were independently repeated three times. One supplementary experiment (Fig. S1) was only performed twice. Information on sample size is given in the figure legends. Definitions on biological and technical replicates are also given in the figure legends where necessary. We have not removed any outliers from any of our experiments.

Sequencing raw data files can be found at <https://www.ebi.ac.uk/ena/browser/view/PRJEB35110>

**Statistical reporting**

- Statistical analysis methods should be described and justified
- Raw data should be presented in figures whenever informative to do so (typically when N per group is less than 10)
- For each experiment, you should identify the statistical tests used, exact values of N, definitions of center, methods of multiple test correction, and dispersion and precision measures (e.g., mean, median, SD, SEM, confidence intervals; and, for the major substantive results, a measure of effect size (e.g., Pearson's r, Cohen's d)
- Report exact p-values wherever possible alongside the summary statistics and 95% confidence intervals. These should be reported for all key questions and not only when the p-value is less than 0.05.

Please outline where this information can be found within the submission (e.g., sections or figure legends), or explain why this information doesn’t apply to your submission:

Information on statistical tests is given in the figure legends. p-values (usually P<0.05) are indicated by asterisks for key experiments in the relevant figures.

(For large datasets, or papers with a very large number of statistical tests, you may upload a single table file with tests, Ns, etc., with reference to sections in the manuscript.)

**Group allocation**

- Indicate how samples were allocated into experimental groups (in the case of clinical studies, please specify allocation to treatment method); if randomization was used, please also state if restricted randomization was applied
- Indicate if masking was used during group allocation, data collection and/or data analysis

Please outline where this information can be found within the submission (e.g., sections or figure legends), or explain why this information doesn’t apply to your submission:

This does not apply to our manuscript.

**Additional data files (“source data”)**

- We encourage you to upload relevant additional data files, such as numerical data that are represented as a graph in a figure, or as a summary table
- Where provided, these should be in the most useful format, and they can be uploaded as “Source data” files linked to a main figure or table
- Include model definition files including the full list of parameters used
- Include code used for data analysis (e.g., R, MatLab)
- Avoid stating that data files are “available upon request”

Please indicate the figures or tables for which source data files have been provided:

Our data (except the structural and genome sequence data) do not represent numerical data and also do not have undergone intensive processing. Sequencing raw data are available in the above mentioned data repository. Structural data are available at PDBe with accession code 6TIF.

We currently believe that providing raw data files for growth curves and protein decay curves would not provide a benefit for the reader. However, if the editors and reviewers would identify experiments, for which inclusion of raw data would be desired, we would be happy to provide the raw data files such as excel data files (for growth curves or protein degradation curves) or the raw images (for Western blots and microscopic pictures).
